# From Micropores to Ultra-micropores inside Hard Carbon: Toward Enhanced Capacity in Room-/Low-Temperature Sodium-Ion Storage

**DOI:** 10.1007/s40820-020-00587-y

**Published:** 2021-03-30

**Authors:** Jinlin Yang, Xiaowei Wang, Wenrui Dai, Xu Lian, Xinhang Cui, Weichao Zhang, Kexin Zhang, Ming Lin, Ruqiang Zou, Kian Ping Loh, Quan-Hong Yang, Wei Chen

**Affiliations:** 1 Joint School of National University of Singapore and Tianjin University, International Campus of Tianjin University, Binhai New City, Fuzhou, 350207 People’s Republic of China; 2 Department of Chemistry, National University of Singapore, 3 Science Drive 3, Singapore, 117543 Singapore; 3 National University of Singapore (Suzhou) Research Institute, 377 Lin Quan Street, Suzhou Industrial Park, Suzhou, 215123 Jiangsu China; 4 Department of Physics, National University of Singapore, 2 Science Drive 3, Singapore, 117542 Singapore; 5 Nanoyang Group, State Key Laboratory of Chemical Engineering, School of Chemical Engineering and Technology, Tianjin University, Tianjin, 300350 People’s Republic of China; 6 Beijing Key Laboratory for Theory and Technology of Advanced Battery Materials, Department of Materials Science and Engineering, College of Engineering, Peking University, Beijing, 100871 People’s Republic of China; 7 Institute of Materials Research and Engineering (IMRE), Agency of Science, Technology, and Research (A*STAR), 3 Research Link, Singapore, 117602 Singapore

**Keywords:** Carbon anode, Ultra-micropores, Extra sodium-ion storage sites, Low-voltage capacity, High areal capacity

## Abstract

**Highlights:**

- Hard-carbon anode dominated with ultra-micropores (< 0.5 nm) was synthesized for sodium-ion batteries via a molten diffusion–carbonization method.
- The ultra-micropores dominated carbon anode displays an enhanced capacity, which originates from the extra sodium-ion storage sites of the designed ultra-micropores.
- The thick electrode (~ 19 mg cm^−2^) with a high areal capacity of 6.14 mAh cm^−2^ displays an ultrahigh cycling stability and an outstanding low-temperature performance.

**Abstract:**

Pore structure of hard carbon has a fundamental influence on the electrochemical properties in sodium-ion batteries (SIBs). Ultra-micropores (< 0.5 nm) of hard carbon can function as ionic sieves to reduce the diffusion of slovated Na^+^ but allow the entrance of naked Na^+^ into the pores, which can reduce the interficial contact between the electrolyte and the inner pores without sacrificing the fast diffusion kinetics. Herein, a molten diffusion–carbonization method is proposed to transform the micropores (> 1 nm) inside carbon into ultra-micropores (< 0.5 nm). Consequently, the designed carbon anode displays an enhanced capacity of 346 mAh g^−1^ at 30 mA g^−1^ with a high ICE value of ~ 80.6% and most of the capacity (~ 90%) is below 1 V. Moreover, the high-loading electrode (~ 19 mg cm^−2^) exhibits a good temperature endurance with a high areal capacity of 6.14 mAh cm^−2^ at 25 °C and 5.32 mAh cm^−2^ at − 20 °C. Based on the in situ X-ray diffraction and ex situ solid-state nuclear magnetic resonance results, the designed ultra-micropores provide the extra Na^+^ storage sites, which mainly contributes to the enhanced capacity. This proposed strategy shows a good potential for the development of high-performance SIBs.
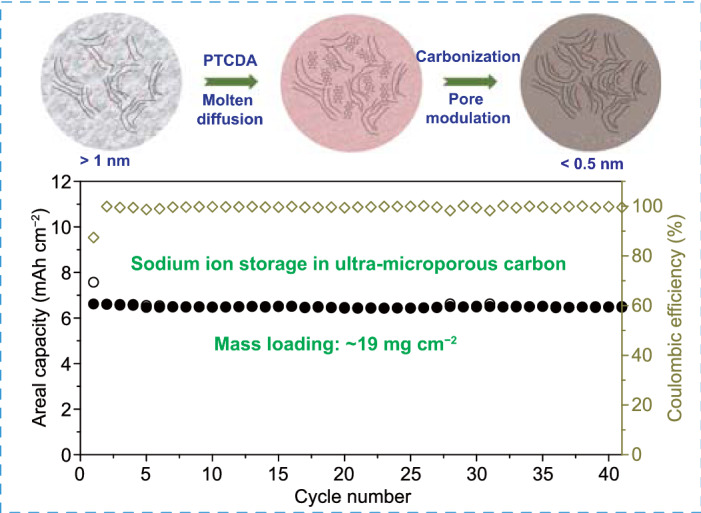

**Supplementary Information:**

The online version of this article (10.1007/s40820-020-00587-y) contains supplementary material, which is available to authorized users.

## Introduction

Sodium-ion batteries (SIBs) have been considered as an important supplement to lithium-ion batteries (LIBs) due to its earth abundancy [1, 2]. Intensive research efforts have been devoted to the development of electrolytes and cathode materials for SIBs [3–6]. High-performance anode materials, which play an equally important role in the further improvement of energy density in SIBs [7, 8], still need rapid development. However, the commercially available graphite anode in LIBs is not suitable for SIBs because of the positive formation energy of Na-graphite intercalation compounds (Na-GIC) [9]. Compared with alloy- and conversion-based anode materials with high plateau voltage and poor cycling stability [10–15], hard carbon has been considered as a promising candidate for the anode materials of SIBs due to its lower plateau voltage and acceptable capacity (~ 300 mAh g^−1^) [16, 17]. Stevens and Dahn demonstrated the feasibility of hard carbon anode in SIBs and proposed a “card-house” model for Na^+^ storage mechanism in glucose-derived hard carbon [18, 19], revealing the intercalation of Na^+^ inside the stacked graphene layers (sloping region) and the pore-filling of Na^+^ into the nanovoids (plateau region). The intercalation and insertion of Na^+^ into the graphitic interlayers and nanovoids of hard carbon were also demonstrated by Komaba et al*.* [20]. Grey et al*.* and Gotoh et al*.* verified that the plateau capacity can be ascribed to the pore filling of Na^+^ into the nanovoids from the ^23^Na solid-state NMR results [21, 22]. However, Cao et al*.* [23] and Qiu et al. [24] elucidated that the plateau capacity originates from the intercalation/deintercalation of Na^+^ into/from the graphitic interlayer spacing larger than 0.37 nm. Ding et al*.* [25], Lotfabad et al. [26], and Alvin et al*.* [27] also attributed the plateau capacity to the intercalation of Na^+^ into the graphitic interlayers. Furthermore, Bommier et al. [28] revealed the linear relationship between sloping capacity and defect concentration and the coexistence of the intercalation and pore-filling process in the plateau region. It has also been suggested that the heteroatom-doping can contribute to the plateau capacity through a systematic first-principles calculation study [29, 30]. However, Zhang et al*. *[31] reported that there is no intercalation in the sloping and plateau region during the sodiation process via* in situ* X-ray diffraction (XRD) measurements for hard carbon.

Low-voltage capacity of anode can enable a higher energy density in full-cell batteries. Therefore, several approaches have been developed to increase the low-voltage capacity. Meng et al*. *[32], Li et al. [33], Lu et al. [34], Zhao et al. [35] , Li et al. [36, 37] used a pore-forming agent or adopted pre-oxidation/high-temperature carbonization to tune the closed pore structure of the hard carbon and hence to realize a large reversible plateau capacity. Choi et al*.* [38] obtained hard carbon fibers with a high plateau capacity by heating waste silk fabrics even at an ultrahigh temperature of 2000 °C. Despite of the aforementioned advances in the synthesis of hard carbon with enhanced reversible plateau capacity, a high carbonization temperature (much higher than 1300 °C) imposes a difficulty for practical applications. In addition, a poor rate capability always accompanies with the high plateau capacity. Therefore, it is essential to develop hard carbons with designed structures under a mild temperature, and to realize a large plateau capacity and satisfying rate capability.

Disordered porous carbon, such as biomass-derived carbon [39], and porous coordination polymer-derived carbon [40], can be readily synthesized under a mild temperature lower than 1000℃. These porous carbon materials deliver improved diffusion kinetics and a satisfying rate capability in SIBs, originating from the existence of the well-developed porosity [41–43]. However, their discharge/charge profiles are dominated by sloping curves, due to the capacitive ion adsorption/desorption on the surface sites of the micropores, where the bare Na^+^ and solvated Na^+^ co-exist [44, 45]. It is found that the existing electrolyte has a significant influence on the ionic interaction inside the pore [46]. If the electrolyte can be prevented to diffuse into the micropores, a different storage mechanism from the capacitive adsorption can be introduced. As previously reported, the aperture size of carbon materials displays an ionic-sieving effect on the solvated Na^+^ and the pore size has a great influence on the electron distribution inside the pore [46–50]. Specifically, the desolvation will happen around the aperture when the aperture size is smaller than the solvated Na^+^ [47–49, 51–53]. In addition, according to the previous theoretical results, the Na^+^ concentration inside the pore increases with the decreasing pore inner diameter [46, 50]. Moreover, the electrons tend to be spread out over all neighboring Na^+^ instead of a single Na^+^ inside the pore with the decreasing pore inner diameter [46]. The tendency of Na^+^ clustering inside the pores thereby becomes feasible. Given that the micropores (> 1 nm) in porous carbon can be transformed to ultra-micropores with a smaller aperture size and pore inner diameter, pore-filling and clustering of bare Na^+^ instead of slovated Na^+^ can be introduced and the fast diffusion of Na^+^ can still be ensured during the sodiation/desodiation process. Besides, the exclusion of solvent insides the pores can ensure more Na^+^ storage sites, which is beneficial to enhance the capacity. Therefore, tuning the aperture size and pore inner diameter of porous carbon shows great potential to significantly improve the capacity without sacrificing the rate capability.

In this work, we developed an approach to effectively increase ultra-micropores inside the carbon materials through molten diffusion of aromatic hydrocarbons into the microporous carbon followed by further carbonization. The optimized carbon anode displays an enhanced capacity of 346 mAh g^−1^ at 30 mA g^−1^ with most of capacity (~ 90%) below 1 V, which is beneficial for the high working voltage of a practical full-cell battery. Moreover, the high-loading electrode (~ 19 mg cm^−2^) also exhibits a high areal capacity of 6.14 mAh cm^−2^ at 25 °C and 5.32 mAh cm^−2^ at − 20 °C, revealing a good temperature endurance. Furthermore, the coin-type full battery enables a high capacity of ~ 97.1 mAh g^−1^. The proposed molten diffusion–carbonization method is facile and energy-efficient to prepare high-performance carbon anode materials with great practical potential for SIBs.

## Experimental Section

### Chemicals and Materials

Activated carbon (AC, XFP06) and cubic structure of mesoporous carbon (CMK-8, XFP02) were purchased from Jiangsu XFNANO Materials Tech Co., Ltd. Perylene-3,4,9,10-tetracarboxylic dianhydride (PTCDA) and potassium hydroxide (KOH, AR, pellets,  ≥ 85%) were obtained from SIGMA-ALDRICH PTE LTD. All the chemicals used throughout this work were used as received without any further purification.

### Synthesis of GC

The dried PTCDA was heated to 900 °C in argon at a rate of 5 °C min^−1^ and then maintained for 5 h. The furnace was then cooled to room temperature at a rate of 5 °C min^−1^. The obtained product was denoted as GC.

### Synthesis of AC + GC

AC and GC powders with a mass ratio of 1:1 was mechanically mixed completely, which is denoted as AC + GC.

### Synthesis of AC1050

The AC powder was heated to 1050 °C in argon at a rate of 5 °C min^−1^ and then maintained for 5 h. The furnace was then cooled to room temperature at a rate of 5 °C min^−1^. The obtained product was denoted as AC1050.

### Synthesis of ACGCx

The AC and PTCDA were vacuum-dried overnight at 110 °C before use. The dried AC and PTCDA were mechanically mixed. The mass ratio of the AC and PTCDA was calculated according to the pore volume of AC and the density of PTCDA (e.g., for 1.391 cm^3^ g^−1^ pore volume of AC, the mass ratio of the AC and PTCDA is about 1:2.36). The PTCDA was then encapsulated into the AC via molten diffusion–carbonization method in argon at 400 °C for 3 h, followed by further carbonization at a specific temperature for another 5 h. Subsequently, the tube furnace was cooled down to room temperature at a rate of 5 °C min^−1^. The obtained products carbonized at different temperature (i.e., 750, 900, 1050, and 1200 °C) were demoted as ACGC*x* (*x* = 750, 900, 1050, 1200).

### Synthesis of ACGC, CMK8GC, HCGC, and LCGC

For hydrothermal reactions, 6.4 g sugar was dissolved into 40 mL water and heated at 180 °C for 8 h. After drying, the obtained black powder (denoted as BP) was carbonized in Ar at 900 °C for 5 h at a rate of 3 °C min^−1^, which was denoted as LC (low-specific-surface-area arbon). For the synthesis of HC (high-specific-surface-area carbon), the dried BP was carbonized in Ar at 500 °C for 2 h at a rate of 5 °C min^−1^. Then the obtained product was physically mixed with KOH with a mass ratio of 1:4. The mixture was activated at 800 °C for 2 h at a rate of 5 °C min^−1^. The synthesis of ACGC, CMK8GC HCGC, and LCGC are similar with that of ACGC1050.

### Characterizations

Scanning electron microscopy (SEM, JSM6700F) and transmission electron microscopy (TEM, JEOL-2010) were adopted to characterize the electrode material. XRD patterns were collected from a Bruker D8 Advance X-ray diffractometer with Cu Kα radiation of 1.5418 Å. The Raman spectra were collected on a Horiba Jobin Yvon Modular Raman Spectrometer at 514 nm (Green). The pore structures were investigated via N_2_ adsorption–desorption isotherms on a NOVA 2200e. The CO_2_ adsorption test was conducted on an Autosorb-iQ. ^23^Na Magic Angle Spinning Nuclear Magnetic Resonance (MAS NMR) experiments was recorded on a Bruker Advance III 400 NMR spectrometer equipped with a superconducting magnet (89 mm wide-bore 9.4 T) and a HX probe (1.3 mm) at a Larmor frequency of 105.8 MHz. Single pulses were applied in acquiring ^23^Na NMR data at a spinning speed of 80 kHz. ^23^Na chemical shifts was referenced to 1 M NaCl solution. True density was measured via an AccuPyc II 1340 analyzer with Helium as analysis gas.

### Electrochemical Measurements

CMCNa binder was first dissolved into DI water to form a uniform binder solution with a concentration of 12.5 mg mL^−1^. The vacuum-dried active material was subsequently added into the binder solution with a weight ratio of 90:10 for active material and binder. The slurry was stirred overnight and then pasted onto copper foil, followed by drying at 50 °C for 4 h. Circular electrodes were obtained via a punch machine and then vacuum-dried at 120 °C for 12 h. The mass loading of the electrode was ~ 1.5–2.0 mg cm^−2^. The coin-type cells (2032) were assembled in an argon-filled glove box, with concentrations of moisture and oxygen below 0.2 ppm. Sodium metal was applied as the counter electrode. A Whatman GF/B glass fiber was adopted as the separator, and the electrolyte was a 1 M sodium triflate (NaOTf) in diethylene glycol dimethyl ether (DEGDME). Galvanostatic discharge/charge results were collected from LAND-CT2001A galvanostats (Wuhan, China) in a range of 0.001–3.0 V (vs. Na/Na^+^) at room temperature. CV cycling were conducted in a range of 0.001–3.0 V at a scan rate of 0.1 mV s^−1^ on an AUTOLAB electrochemical workstation. The full cells were assembled using a sodiated LCGC anode and PTCDA cathode or LCGC anode and a sodiated PTCDA cathode with a weight ratio of 1:2.54. The full cell was cycled in the range of 0.5–3.0 V.

## Results and Discussion

### Synthesis and Characterization of the Ultra-micropore Dominated Carbon

**Fig. 1:**
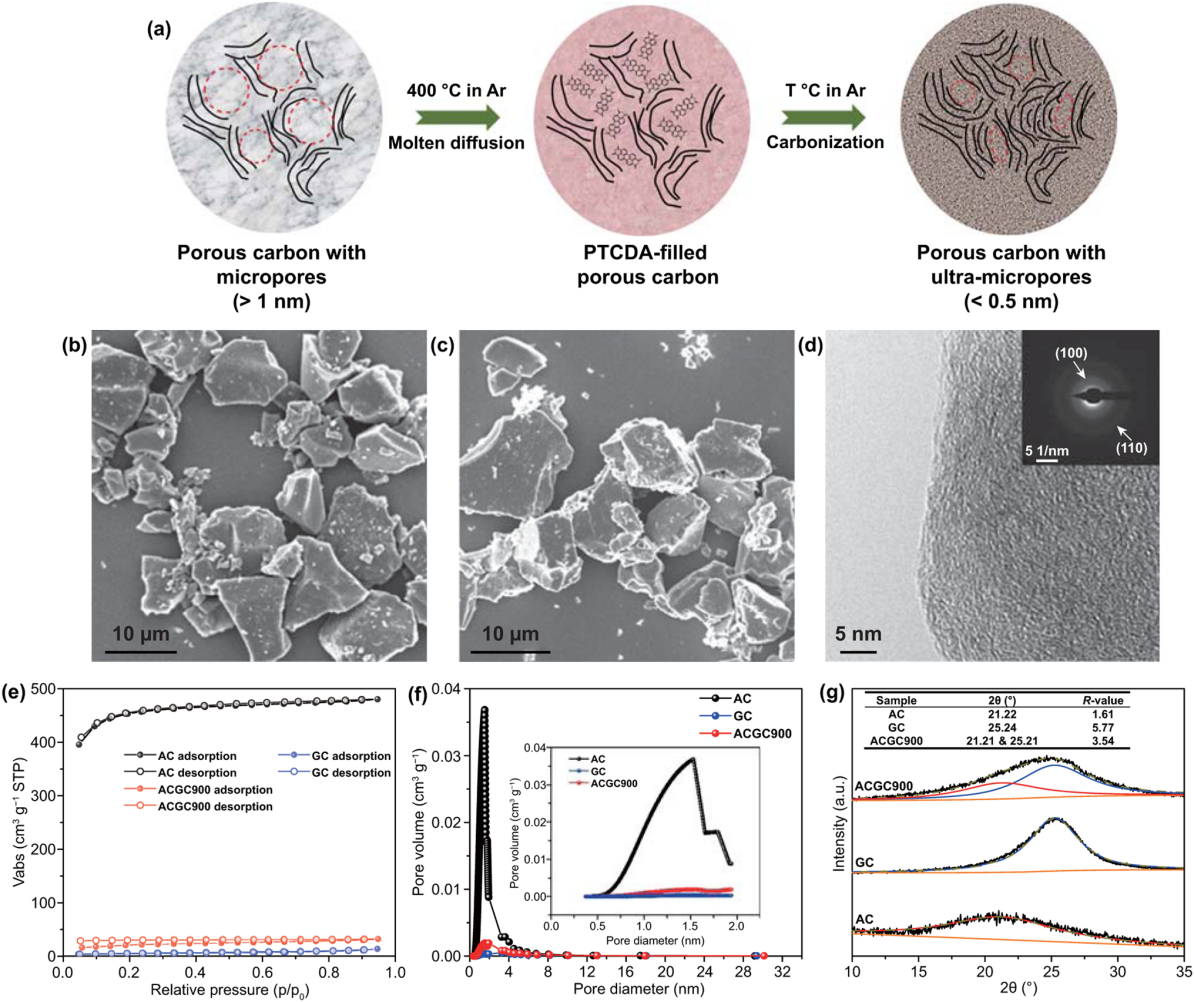
Characterizations of the AC, GC and ACGC900. **a** Scheme of the molten diffusion–carbonization strategy (T represents the carbonization temperature). SEM image of **b** AC, and **c** ACGC900. **d** TEM image of ACGC900 and corresponding selected area electron diffraction (SAED) pattern (inset). **e** N_2_ adsorption/desorption isotherm, **f** pore size distribution from N_2_ adsorption/desorption measurement and **g** peak fitting of the (002) peaks in the XRD patterns

A facile, cost-effective, and massive production process was developed to synthesize ultra-micropores (< 0.5 nm) dominated hard carbon via a molten diffusion–carbonization method (Fig. 1a). Microporous carbon and perylenetetracarboxylic dianhydride (PTCDA) were first mechanically blended with a specific mass ratio and then annealed at 400 °C in argon for 3 h. Above the melting point (~ 350 °C) of PTCDA, the molten PTCDA diffused into the microporous carbon and subsequently adsorbed on the inner surface of micropores [54]. During the further carbonization process, PTCDA inside the micropores was carbonized, while the residual PTCDA was evaporated and released with the argon flow. Commercial activated carbon (AC) was selected as a model host due to its developed porosity.

After the molten diffusion-carbonization process, the morphology of the micron-sized AC particles is maintained well without obvious residual of PTCDA-derived carbon, as revealed by SEM images in Figs. 1b, c and S1. TEM images of the designed carbon, ACGC900 (the related abbreviations can be seen in the Sect. 2), reveal a disordered structure without obvious porosity, which is different from the microporous structure of AC (Figs. 1d and S2). The selected area electron diffraction (SAED) pattern of ACGC900 particle indicates a certain degree of graphitization with an obvious reflection ring corresponding to the (110) planes of graphitic structure, which can be attributed to the introduction of PTCDA-derived carbon inside the micropores (inset in Fig. 1d). Moreover, the Brunauer–Emmett–Teller (BET) specific surface area (S_BET_) is greatly reduced from 1429 m^2^ g^−1^ for AC to 48.4 m^2^ g^−1^ for the designed ACGC900. Compared with AC, the micropores with a size of ~ 1.5 nm cannot be detected in ACGC900 (Fig. 1f), suggesting that most of the micropores are modulated to be ultra-micropores inaccessible by N_2_. Besides, the effect of the carbonization on the structure of AC is also explored (Fig. S3 and Table S2), indicating no obvious structure evolution. Notably, as shown in Figs. 1e and S12, there exists an obvious hysteresis between the N_2_ adsorption/desorption isotherms of ACGC*x* (*x* = 750, 900, 1050, and 1200), indicating the existence of restricted pores or ultra-micropores [18]. Due to the smaller molecular size (3.3 Å for CO_2_ vs 3.64 Å for N_2_) and the higher working temperature (273 K for CO_2_ vs 77 K for N_2_), CO_2_ adsorption measurement is highly efficient to detect the existence of ultra-micropores (< 0.5 nm) [55]. As displayed in Fig. S4 and Table S3, the S_BET_ of ACGC900 calculated from CO_2_ adsorption measurement is ~ 196.6 m^2^ g^−1^, much higher than that calculated from N_2_ adsorption/desorption. Moreover, the pore size of ACGC900 is mainly distributed at 0.3–0.5 nm, along with a pore volume of ~ 0.365 cm^3^ g^−1^. This reveals the existence of numerous ultra-micropores in ACGC900, which can reduce the interfacial contact between the inner surface of carbon and electrolyte and thus minimize side reactions.

The microstructure of the rationally designed carbon was further investigated via XRD measurements (Fig. 1g). Compared with that of AC, the (002) peak of ACGC900 becomes broader and shifts to a higher degree. By applying the profile-fitting method, the broad peak of ACGC900 can be divided into two parts [56]. Specifically, the fitted peak located at around 25.21° and 21.21° can match well with the (002) peak of the GC and AC, respectively (inset table in Fig. 1g). The coexistence of two different carbon phases further demonstrates the successful encapsulation, consistent with the SEM results (Fig. 1b, c). The *R* value, an indicator of graphitization degree in carbon materials, is also calculated from XRD patterns (Fig. S5) [57]. As displayed in the inset table of Fig. 1g, the *R* value of ACGC900 is higher than that of AC, indicating a higher graphitization degree for ACGC900, in good accordance with the Raman results (Fig. S6) [57]. The improved graphitization can ensure a good electroconductivity and hence enable a fast discharge/charge capability.

### Electrochemical Performance in Sodium-Ion Batteries

**Fig. 2:**
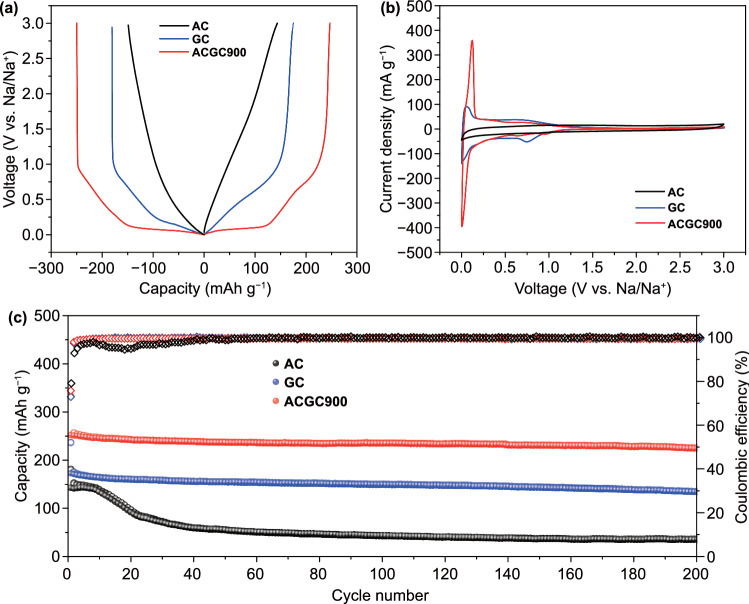
Electrochemical performance of the AC, GC and ACGC900 electrode in SIBs: **a** Galvanostatic discharge/charge curves at 50 mA g^−1^. **b** CVs in a voltage range of 0.001–3.0 V vs Na/Na^+^ under 0.1 mV s^−1^. **c** Cycling performance at 50 mA g^−1^

The electrochemical properties of AC, GC, and ACGC900 electrodes were evaluated by galvanostatic discharge/charge measurements. In Fig. 2a, the discharge/charge profiles of the AC and GC electrodes display all sloping curves in the voltage range of 0.001–3 V, which mainly result from the capacitive storage of Na^+^ on surface sites [49, 58]. For ACGC900 electrode, an apparent plateau appears at ~ 0.1 V along with the sloping region between 0.1 and 1 V. Accordingly, a couple of redox peaks at ~ 0.1 V are also present in the cyclic voltammetry (CV) curves (Figs. 2b and S7). Moreover, the Na^+^ storage behavior in the carbonized AC electrode keeps the same with that in the pristine AC electrode (Fig. S8), demonstrating the significant role of the molten-diffusion process. To further demonstrate the advantage of the designed structure, the mechanically mixed AC + GC (mass ratio of AC and GC is 1:1) electrode was also fabricated. As shown in Fig. S9, nearly no redox peaks can be observed in the CV curves of the AC + GC electrode, suggesting the significance of the molten diffusion process. As the sloping capacity of ACGC900 electrode is nearly equal to that of AC electrode, the increased capacity originating from the plateau region can be attributed to the extra storage sites from the ultra-micropores. Furthermore, the cycling performance and rate capability of these three electrodes were compared in Figs. 2c and S10. When AC electrode was cycled at 50 mA g^−1^, sharp drop of capacity can be observed from the 10^th^ cycles onward and nearly no capacity can be obtained after 200 cycles. The rapid failure of AC electrode could be ascribed to the high accessible surface area and low electroconductivity, which can lead to increased solid electrolyte interface (SEI) layers [59, 60]. While for ACGC900 electrode, ~ 90% initial capacity can be reserved after 200 cycles. The improved cycling stability could be ascribed to the structure after the encapsulation, including the reduced interfacial contact between the electrode and electrolyte and the increased degree of graphitization [61]. Besides, more than 100 mAh g^−1^ can be obtained for the ACGC900 electrode at 2000 mA g^−1^ while nearly no capacity for the AC electrode, further demonstrating the advantage of the designed structure after the molten diffusion–carbonization process.

**Fig. 3:**
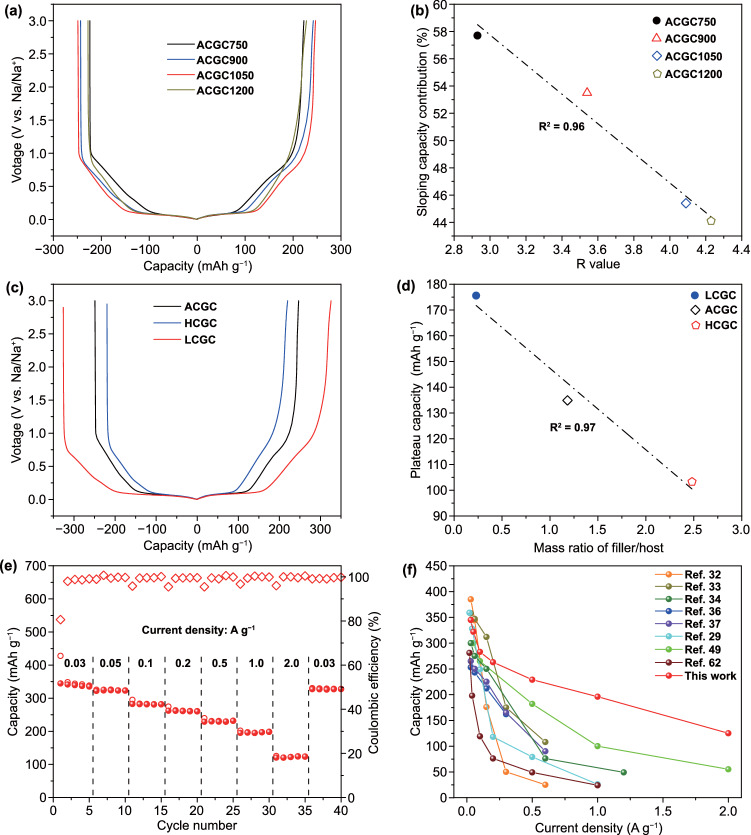
Effects of carbonization temperature and pore volume on the electrochemical performance: **a** Galvanostatic discharge/charge curves of ACGCx at 50 mA g^−1^, and **b** the relationship between the sloping capacity contribution and the calculated ***R*** value from XRD. **c** Galvanostatic discharge/charge curves of ACGC, HCGC, LCGC electrodes at 50 mA g^−1^. **d** The relationship between the plateau capacity and the mass ratio of the filler/host. **e** rate performance and **f** the comparison of the rate performance of LCGC anode with that of hard carbon anodes recently reported in SIBs

To further explore the relationship between the microstructure and sodium-ion storage performance, ACGC*x* (*x* = 750, 900, 1050, and 1200) samples were prepared under various carbonization temperatures. The morphology of ACGCx can be found in Fig. S11. Besides, CO_2_ and N_2_ adsorption/desorption measurements were conducted to explore the evolution of pore structure in ACGCx (Figs. S12 and S13), which are summarized in Tables S1 and S3. As shown in Tables S1 and S3, the S_BET_ of ACGCx decreases with the increasing temperature. Moreover, the skeletal (true) density monotonically increases from 1.89 (ACGC750) to 2.14 g cm^−3^ (ACGC1200) as the temperature elevated, which is obtained from helium pycnometry measurement. These temperature-dependent properties suggest the formation of more ultra-micropores in ACGCx with increasing temperature. Figure 3a displays the discharge/charge curves of ACGCx electrodes at 50 mA g^−1^ with a voltage range of 0.001–3 V. The increasing plateau capacity/sloping capacity ratio with the increased temperature indicates more Na^+^ storage sites generating from the ultra-micropores, which is consistent with the results from helium pycnometry test and CO_2_ adsorption measurement. The interlayer spacing also evolves with the carbonization temperature. As shown in Fig. S14 and its inset table, the interlayer spacing decrease from 0.362 to 0.350 nm with the increasing carbonization temperature, while the plateau capacity does not decrease accordingly. This suggests that the intercalation is not the origin of the plateau capacity. On the contrast, the sloping capacity contribution decreases linearly with the increasing ***R*** value of ACGCx (Figs. 3b, S14–S16 and Table S4), where a lower ***R*** value suggests a lower degree of graphitization or more defect sites [28, 32]. The decreasing sloping capacity with increasing temperature could be attributed to the reduced defect sites, which can be further demonstrated by the relationship between the sloping capacity contribution and the *I*_D_/*I*_G_ ratio obtained from Raman spectra (Figs. S17, S18). Moreover, the initial coulombic efficiency (ICE) value increases linearly with the decreasing S_BET_ from N_2_ adsorption/desorption measurement (Fig. S19 and Table S5), indicating reduced parasitic reactions toward electrolyte [61, 62].

The ACGC1050 electrode displays the highest plateau capacity and whole capacity among all ACGCx electrodes, along with the best rate performance and cycling stability (Fig. S20). Therefore, the optimized temperature is determined to be 1050 °C. Furthermore, the relationship between the pore structure of the porous carbon host and the electrochemical performance was also studied at the optimized temperature (Figs. S21–S24 and Table S6). Specifically, LC (low S_BET_ carbon, 224 m^2^ g^−1^) and HC (high S_BET_ carbon, 2939 m^2^ g^−1^) were also selected as carbon host for comparison. After the molten diffusion–carbonization process, the obtained LCGC and HCGC materials suffer from huge reduction in specific surface area, which is in consistent with ACGC (Table S7). Besides, the skeletal (true) density of LCGC, ACGC, and HCGC are also compared in Table S7, suggesting various ultra-micropore volumes existing in these carbon materials. As shown in Figs. 3c and S25, the LCGC electrode delivers the highest capacity among these carbon electrodes and a stable cycling performance. A quantitative relationship is built between the plateau capacity and the mass ratio of the filler/host, *i.e.,* a lower filler/host ratio corresponding to more ultra-micropores can ensure a larger plateau capacity (Fig. 3d and Table S7). Therefore, porous carbon host with micropore-dominated structure and low S_BET_ can enable more plateau capacity at the optimized temperature of 1050 °C. Apart from a high reversible capacity of 346 mAh g^−1^ at 30 mA g^−1^, the LCGC electrode also demonstrates a satisfying rate capability. As shown in Fig. 3e, a high reversible capacity of 125 mAh g^−1^ can still be delivered at 2000 mA g^−1^, which reveals a superior performance compared to that of the previously reported hard carbon anode materials (Fig. 3f and Table S8) [29, 32–34, 36, 37, 49, 62].

### Sodium-Ion Storage Mechanism

**Fig. 4:**
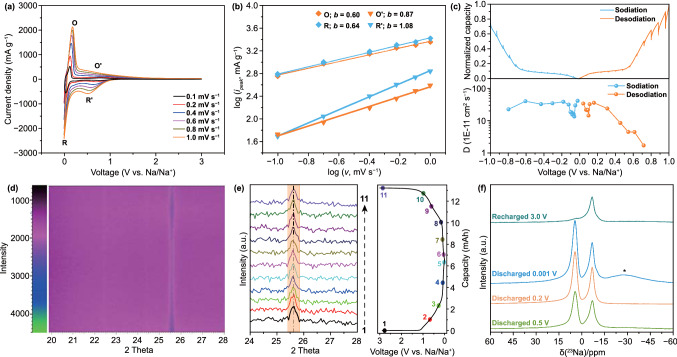
Analysis of sodium-ion storage mechanism: **a** CV curves under various scan rates from 0.1 to 1.0 mV s^−1^, **b** the corresponding linear fitting of the plots between log(*i*) and log(*v*) and **c** *D*_Na_^+^ values calculated from GITT measurement during discharge/charge process. **d, e** The in situ XRD mapping with the capacity-potential curve under voltage windows of 0.001–3 V. **f** The ex situ solid-state NMR spectra of ^23^Na at different potentials

We propose that the introduction of ultra-micropores leads to the plateau capacity. To demonstrate the proposal, scan-rate-dependent CV from 0.1 to 1.0 mV s^−1^ and the galvanostatic intermittent titration technique (GITT) measurements were further conducted (Figs. 4a–c and S26–S28). During the discharge/charge process, both surface capacitive adsorption and diffusion-controlled process contribute to the sodium-ion storage. The contribution ratio of the two mechanisms can be quantitatively determined by the power-law formula: *i* = *av*^*b*^. As displayed in Fig. 4b, the *b*-values of the high-voltage sloping region in ACGC electrode are ~ 1.0, corresponding to a fast adsorption/desorption of Na^+^ at the surface-active sites [63, 64]. However, the *b* values of the low-voltage plateau region in the ACGC electrode are ~ 0.5, suggesting a diffusion-controlled process [65]. Meanwhile, the profiles of the Na^+^ diffusion coefficient (*D*_Na_^+^) as a function of potential reveal a U-turn point at ~ 0.05 V during the discharge/charge process (Fig. 4c). The rapid drop of *D*_Na_^+^ at ~ 0.05 V could be ascribed to the large diffusion barrier for Na^+^ insertion into the ultra-micropores, consistent with the low *b*-values of the low-voltage plateau region (Fig. 4b). The reverse increment of *D*_Na_^+^ near the cutoff voltage corresponds to the adsorption and clustering of the Na^+^ inside the ultra-micropores. Therefore, the adsorption of Na^+^ on the surface sites is a capacitive behavior, whereas the insertion of Na^+^ into the ultra-micropores is a diffusion process.

*In situ* XRD measurement is an effective and real-time technique to detect the possible variation of interlayer spacing during the discharge/charge process. Hence, the *in situ* XRD pattern of the second discharge/charge cycle at 0.15 mA cm^−2^ was collected for analysis. As shown in Fig. 4d, the band located at 25.6° represents the (002) peak, suggesting a d-spacing of ~ 3.5 Å. If intercalation exists, (002) peak detected from XRD pattern will experience a peak shifting during the discharge/charge process [66]. From Fig. 4d, e, no peak shift of the (002) peak or new peak can be observed during the whole process, suggesting no intercalation/deintercalation into/from the graphitic interlayers in both sloping region and plateau region. Raman spectra of the carbon electrode at various states were further collected to reveal any possible peak shift or new peaks during the sodiation/desodiation process. As shown in Fig. S29, the D-band (1340 cm^−1^) and G-band (1580 cm^−1^) keep the same peak location and no new peaks appear during the sodiation/desodiation process, suggesting no intercalation of Na^+^ into/from graphitic interlayers [33]. Along with the relationship between the sloping capacity and ***R*** value or *I*_D_/*I*_G_ ratio (Figs. 3b and S18), in situ XRD results further corroborate that the sloping capacity results from the adsorption/desorption of Na^+^ to/from the surface sites, which is also in accordance with the high *b*-values of the sloping region.

To further explore the sodium-ion storage mechanism in the plateau region, ex situ ^23^Na MAS NMR measurements were conducted. As shown in Figs. 4f and S30, two resonances at − 6.87 and 4.44 ppm can be observed in the spectra obtained from the electrode discharged to 0.5 and 0.2 V. Note that there is no washing process for the electrode, the peak at − 6.87 ppm can be ascribed to the sodium salt in the electrolyte. Along with discharging, the appearance of the peak at 4.44 ppm indicates the adsorption of Na^+^ on the surface sites [21]. When the electrode was fully discharged to 0.001 V, a broad peak located between − 20 and − 30 ppm appears, indicating the existence of Na^+^ with a more restricted mobility, e.g., in ultra-micropores [67]. When the electrode is recharged to 3 V, only the peak at − 6.87 ppm retains and other peaks disappear accordingly, suggesting the reversible storage of Na^+^ on the surface and inside the ultra-micropores. Therefore, it is indeed the pore-filling of Na^+^ into the ultra-micropores that contribute to the low-voltage plateau at ~ 0.1 V [46]. Figure S31 schematically illustrates the aforementioned process in a sodiation process.

### Electrochemical Performance of Thick Electrodes and Full-cell Batteries

**Fig. 5:**
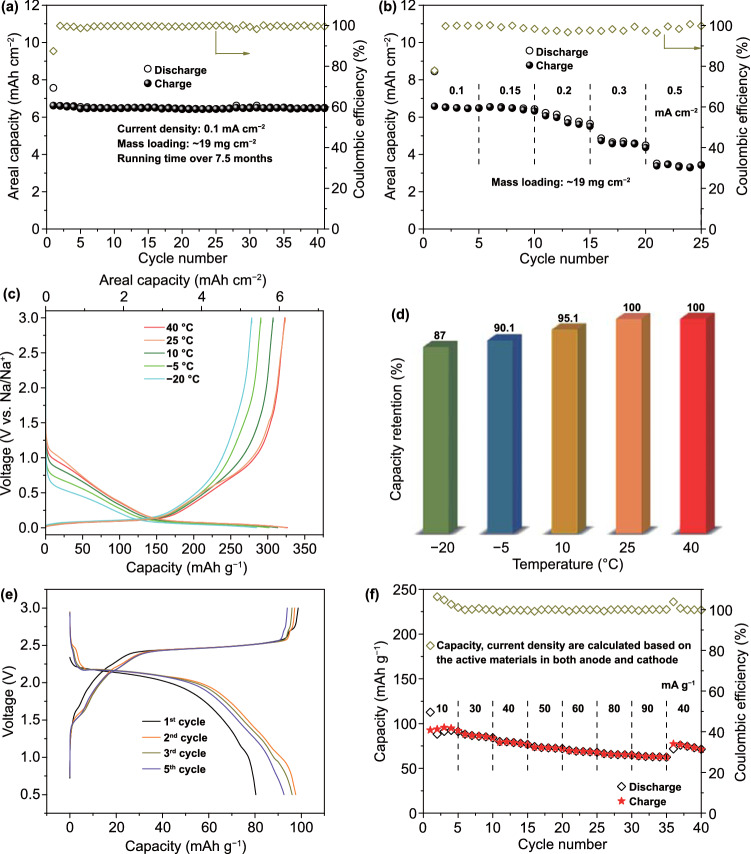
Thick electrode and full-cell test. **a** Cycling performance, and **b** rate capability of the thick electrode with a mass loading of ~ 19 mg cm ^−2^. **c** galvanostatic discharge/charge curves at 0.2 mA cm ^−2^ in the temperature range from − 20 to 40 °C and **d** the capacity retentions of thick electrode. **e** Galvanostatic discharge/charge curves at 10 mA g^−1^ and **f** rate capability of full cell in the voltage range of 0.5–3.0 V

For the practical applications, excellent sodium-ion storage performance under room-/low-temperature in thick electrode is crucial. Hence, a thick electrode of LCGC with a mass loading of ~ 19 mg cm^−2^ was further fabricated. As shown in Fig. 5a, a high areal capacity of 6.14 mAh cm^−2^ can be achieved at 0.1 mA cm^−2^. After 41 cycles (~ 7.5 months cycling), nearly no capacity degradation can be observed, indicating an excellent cycling stability of this electrode even with a high mass loading. Compared with the high reversible capacity at a small current density (0.1 mA cm^−2^), ~ 53.1% of capacity (3.26 mAh cm^−2^) can still be maintained at a much higher current density (0.5 mA cm^−2^), indicating the superior rate capability of the fabricated thick electrode (Figs. 5b and S32). As shown in Fig. 5c, the low-temperature performance of the thick LCGC electrode was further explored from − 20 to 40 °C. Specifically, the capacity retentions of the thick electrode are around 87, 90, 95, 100, and 100% compared to the capacity obtained at 25 °C, respectively (Fig. 5d). Moreover, similar diffusion kinetics of Na^+^ inside the thick electrode at various temperatures can be observed from the potential-dependant *D*_Na_^+^ profiles in Fig. S33, suggesting a satisfying low-temperature performance. Furthermore, the electrochemical performance was evaluated for a coin-type full battery. The LCGC anode and a sodiated organic cathode (PTCDA) were assembled into the full battery with a N/P ratio (the areal capacity ratio of negative to positive electrode) of 1.15:1. Figure 5e  displays the initial five discharge/charge cycles of the full cell at 10 mA g^−1^ within a range of 0.5–3.0 V. A high reversible capacity of ~ 97.1 mAh g^−1^ can be obtained. It is worth noting that the current density and the capacity are calculated from the total mass of the active materials. The rate capability of the full-cell battery was also evaluated via assembling a sodiated LCGC anode with the PTCDA cathode. When the current density is increased to 90 mA g^−1^, a reversible capacity of 63.6 mAh g^−1^ can be reserved with a capacity retention ratio of 67.7% (Fig. 5f), indicating an excellent rate capability of the full-cell battery.

## Conclusions

In summary, we have developed a molten diffusion-carbonization strategy to block the micropores of porous carbon and transform them into ultra-micropores (0.3–0.5 nm). The ultra-micropores can only be accessible to the bare Na^+^ without electrolytes, which can effectively minimize the decomposition of electrolytes and then induce a high ICE value of ~ 80.6%. The optimized anode exhibits a good electrochemical performance, i.e., high capacity, superior cycling stability, and satisfying rate performance. With the help of a series of scan-rate-dependent CV, GITT, *in situ* XRD, and ex situ solid-state NMR measurements, it is well demonstrated that the designed ultra-micropores provide the extra sodium-ion storage sites, which mainly contributes to the enhanced capacity. It is noteworthy that the thick electrode with a high areal capacity of 6.14 mAh cm^−2^ displays an ultrahigh cycling stability with a running time over 7.5 months, a satisfying rate capability and an outstanding low-temperature performance. Moreover, the coin-type full battery based on the PTCDA cathode and the sodiated anode possesses a high reversible capacity and superior rate performance. These findings suggest a promising strategy to design practical carbon anode materials for SIBs with high energy density, high rate performance and excellent low-temperature performance.

## Supplementary Information

Below is the link to the electronic supplementary material.

Supplementary Information1 (PDF 3240 kb)
