# Supplementary material for: From Micropores to Ultra-micropores inside Hard Carbon: Toward Enhanced Capacity in Room-/Low-Temperature Sodium-Ion Storage
Source: Nanomicro Lett. 2021 Mar 30;13:98. doi: 10.1007/s40820-020-00587-y (PMC8010088; doi:10.1007/s40820-020-00587-y)
Supplement: Supplementary file 1 — Supplementary Information1 (PDF 3240 kb) [file 40820_2020_587_MOESM1_ESM.pdf]

Supporting Information for

From Micropores to Ultra-Micropores inside Hard Carbon: Towards Enhanced Capacity in Room/Low-Temperature Sodium-Ion Storage

Jinlin Yang[1, 2, 3], Xiaowei Wang[2], Wenrui Dai[2, 3], Xu Lian[2], Xinhang Cui[3, 4], Weichao Zhang[5], Kexin Zhang[6], Ming Lin[7], Ruqiang Zou[6], Kian Ping Loh[2], Quan-Hong Yang[1, 5, *], Wei Chen[1, 2, 3, 4, *]

[1]Joint School of National University of Singapore and Tianjin University, International Campus of Tianjin University, Binhai New City, Fuzhou 350207, P. R. China

[2]Department of Chemistry, National University of Singapore, 3 Science Drive 3, 117543, Singapore

[3]National University of Singapore (Suzhou) Research Institute, 377 Lin Quan Street, Suzhou Industrial Park, Jiangsu, 215123, China

[4]Department of Physics, National University of Singapore, 2 Science Drive 3, 117542, Singapore

[5]Nanoyang Group, State Key Laboratory of Chemical Engineering, School of Chemical Engineering and Technology, Tianjin University, Tianjin, 300350, P. R. China

[6]Beijing Key Laboratory for Theory and Technology of Advanced Battery Materials, Department of Materials Science and Engineering, College of Engineering, Peking University, Beijing 100871, P. R. China

[7]Institute of Materials Research and Engineering (IMRE), Agency of Science, Technology, and Research (A*STAR), 3 Research Link, Singapore 117602, Singapore

*Corresponding authors. E-mail: phycw@nus.edu.sg (W. Chen); qhyangcn@tju.edu.cn (Q-H. Yang)

S1 Supplementary Notes

As shown in Fig. S1, the morphology of GC is obviously different from that of the AC (Fig. 1b). The GC particles are in a rod-like morphology and composed of hierarchical flakes while the AC particles are in a dense chunk-like morphology.

As shown in Fig. S2, transmission electron microscopy (TEM) images of the designed carbon, ACGC900, reveal a disordered structure without obvious porosity, which is different from the microporous structure of AC (Fig. 1d).

Figure S3 shows the exploration about the effect of the carbonization on the structure of AC. As shown in Fig. S3 and Table S2, the pore structure of AC maintains well after carbonization under 1050 °C.

As displayed in Fig. S4 and Table S3, the S_{BET} of ACGC900 calculated from CO_2 adsorption measurement is ~ 196.6 m^2 g^{-1}, much higher than that calculated from N_2 adsorption/desorption. Moreover, the pore size of ACGC900 is mainly distributed at the range of 0.3-0.5 nm, along with a pore volume of ~ 0.365 cm^3 g^{-1}.

Figure S5 displays the method of calculating ***R*** value from the ratio of the (002) Bragg peak intensity to the background signal. ***R*** value (R = B/A) can determine the graphitization degree of the samples. A lower ***R*** value suggests a lower degree of graphitization or less stacked graphene layers.

As displayed in the inset table of Fig. S6, the I_D/I_G value of ACGC900 is lower than that of AC, indicating a higher graphitization degree for ACGC900.

Figure S7 displays the first five consecutive CVs of AC, GC, and ACGC900 in a voltage range of 0.001-3.0 V *vs* Na/Na^+ at a scan rate of 0.1 mV s^{-1}. For ACGC900 electrode, an apparent plateau appears at ~ 0.1 V along with the sloping region above 0.1 V in Fig. 2a. Accordingly, a couple of redox peaks at ~ 0.1 V are also present in the cyclic voltammetry (CV) curves (Figs. 2b and Fig. S7).

Figure S8 shows that the Na^+ storage behavior in the carbonized AC electrode keeps the same with that in the pristine AC electrode, demonstrating the significant role of the molten-diffusion process.

Figure S9 shows the galvanostatic discharge/charge curves at 50 mA g^{-1} and CVs in a voltage range of 0.001-3.0 V vs Na/Na^+ under 0.1 mV s^{-1} of AC, GC, ACGC900, and AC+GC. For ACGC900 electrode, an apparent plateau appears at ~ 0.1 V along with the sloping region above 0.1 V in Fig. 2A. Accordingly, a couple of redox peaks at ~ 0.1 V are also present in the cyclic voltammetry (CV) curves (Figs. 2b and S7). As shown in Fig. S9**,** nearly no redox peaks can be observed in the CV curves of the AC+GC electrode, suggesting the significance of the molten-diffusion process.

As shown in Fig. S10, a capacity of more than 100 mAh g^{-1} can be obtained for the ACGC900 electrode at 2000 mA g^{-1} while nearly no capacity for the AC electrode, further demonstrating the advantage of the designed structure after the molten diffusion-carbonization, including the reduced interfacial contact between the electrode and electrolyte and the increased degree of graphitization.

As revealed by the SEM images in Fig. S11, after the molten diffusion-carbonization process, the morphology of the micron-sized AC particles is maintained well without obvious residual of PTCDA-derived carbon.

As displayed in Fig. S12, the hysteresis between the adsorption and desorption branches of the isotherm indicates the existence of restricted pore. As shown in Table S1 and Table S3, the S_{BET} of ACGCx decreases with the increasing temperature.

As shown in Fig. S13, the size and S_{BET} of micropores decrease with the increasing carbonization temperature, indicating decreased aperture size and pore inner diameter.

As shown in the Fig. S14 and its inset table, the interlayer spacing decrease from 0.362 nm to 0.350 nm with the increasing carbonization temperature.

As shwon in Fig. 3b, Figs. S14-S16 and Table S4, the sloping capacity contribution decreases linearly with the increasing ***R*** value of ACGCx, where a lower ***R*** value suggests a lower degree of graphitization or more defect sites. Besides, the initial coulombic efficiency (ICE) value of various samples ranges from 68.1 to 87.9% (Fig. S16).

As shown in Figs. S17 and S18, the decreasing sloping capacity with increasing temperature could be attributed to the reduced population of defect sites, which can be further demonstrated by the relationship between the sloping capacity contribution and the I_D/I_G ratio obtained from Raman spectra.

Figure S19 shows that the initial coulombic efficiency (ICE) value increases linearly with the decreasing S_{BET} from N_2 adsorption/desorption measurement (Table S5), indicating reduced parasitic reactions towards electrolyte.

As shown in Fig. S20, the ACGC1050 electrode displays the best rate performance and cycling stability. Specifically, the ACGC1050 electrode can reach ~118 mAh g^{-1} even at 2000 mA g^{-1}. Moreover, ~97.3% of the initial capacity can be maintained after 200 cycles at 50 mA g^{-1}.

In Fig. S21, the hysteresis between the adsorption and desorption branches of the isotherm indicates the existence of restricted pores. Besides, the S_{BET} of ACGCx decreases with increasing temperature.

As shown in Figs. S22 and S23, CMK8GC electrode derived from the mesopore-dominated carbon host (CMK-8) displays no plateau capacity during discharge-charge process. This can be explained that the mesopore inside CMK-8 (cubic Ia3d, rod-type) is interconnected and large enough to host the quasi-graphitic nanodomains derived from the filling PTCDA. As a result, nanodomains with layered graphitic structure instead of nanocavity was introduced into the CMK8GC and then no plateau occurs.

As shown in Fig. S24, CMK8GC electrode derived from the mesopore-dominated carbon host (CMK-8) displays no plateau capacity during discharge-charge process.

Figure S25 shows that the ACGC1050 electrode can reach about 118 mAh g^{-1} even at 2000 mA g^{-1}. Moreover, about 97.3% of the initial capacity can be maintained after 200 cycles at 50 mA g^{-1}.

As displayed in Figs. S26-S28, if the cell voltage is linearly proportional to $\tau^{1/2}$, the diffusion coefficient can be calculated from the GITT potential profiles by Fick’s second law with the following equation:

$$\mathrm{D} = \frac{4}{\pi\tau}\left(\frac{m_B V_M}{M_B S}\right)^2\left(\frac{\Delta E_s}{\Delta E_\tau}\right)^2$$

The density of carbon was calculated according to the following equation:

$$\rho = \frac{1}{V_{\mathrm{total}} + \frac{1}{\rho_{carbon}}}$$

where ρ (g cm^{-3}) is the density of carbon, V_{total} (cm^3 g^{-1}) is the total pore volume measured from the N_2 isotherm, ρ_{carbon} is the true density of carbon (2.08 g cm^{-3}).

For the GITT tests, the cell was discharged/charged at C/10 with a current pulse duration of 0.5 h and an interval of 1 h.S1,S2

As shown in Fig. S29, the D-band (1340 cm^{-1}) and G-band (1580 cm^{-1}) keep the same peak location and no new peaks appear during the sodiation/desodiation process, suggesting no intercalation of Na^+ into/from graphitic interlayers.

Figure S30 shows that no resonance peak can be observed at around 750 ppm, indicating no quasi-metallic Na metal existing in the discharged electrode.

Figure S31 schematically illustrates the aforementioned “adsorption/pore-filling” mechanism in a sodiation process, *i.e.* the sloping region above 0.1 V results from the adsorption of Na^+ on the surface sites, while the plateau region below 0.1 V originates from the pore-filling process of Na^+ into the ultra-micropores.

As demonstrated in Fig. S32, compared with the high reversible capacity at a small current density (0.1 mA cm^{-2}), ~ 53.1 % of capacity (3.26 mAh cm^{-2}) can still be maintained at a

much higher current density (0.5 mA cm^{-2}), indicating the superior rate capability of the fabricated thick electrode.

Figure S33 indicates that similar diffusion kinetics of Na^+ inside the thick electrode at various temperatures can be observed from the potential-dependant D_{Na^+} profiles, suggesting a satisfying low-temperature performance.

As shown in Table S1, the specific surface area of ACGCx calculated from N_2 adsorption/desorption test decreases with the increasing carbonization temperature. S_{BET} of ACGCx decreases with increasing temperature.

Table S2 shows the exploration about the effect of the carbonization on the structure of AC. As shown in Fig. S3 and Table S2, the pore structure of AC maintains well after carbonization under 1050 ℃.

Table S3 show the skeletal (true) density data of the ACGCx materials. Specifically, the skeletal (true) density data were recorded on an AccuPyc II 1340 analyzer using Helium as analysis gas. The skeletal (true) density monotonically increases from 1.89 g cm^{-3} (ACGC750) to 2.14 g cm^{-3} (ACGC1200) as the temperature elevated.

Table S4 indicates that the ***R*** value of ACGCx increases with the carbonization temperature, where a lower ***R*** value suggests a lower degree of graphitization or more defect sites.

As shown in Table S5, the initial coulombic efficiency (ICE) value of various samples ranges from 68.1 to 87.9%.

As indicated in Table S6, after the molten diffusion-carbonization process, the obtained ACGC, LCGC, HCGC and CMK8GC and HCGC materials suffer from a huge reduction in specific surface area as compared with AC, LC, HC, and CMK8, respectively.

As shown in Table S7, LC (low S_{BET} carbon, 224 m^2 g^{-1}) and HC (high S_{BET} carbon, 2939 m^2 g^{-1}) were also selected as carbon host for comparison. After the molten diffusion-carbonization process, the obtained LCGC and HCGC materials suffer from a huge reduction in specific surface area, which is consistent with ACGC.

As shown in Table S8, a high reversible capacity of 125 mAh g^{-1} can still be delivered at 2000 mA g^{-1}, which reveals a superior performance compared to that of the previously reported hard carbon anode materials.S3-S13

S2 Supplementary Figures and Tables

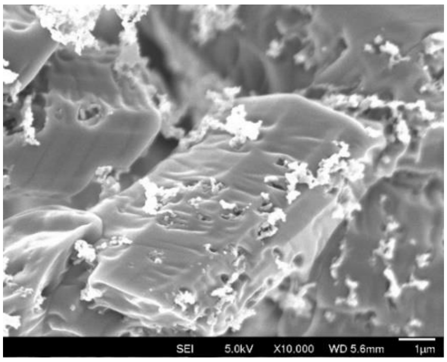

Fig. S1 SEM image of GC (Scale bar: 1 μm)

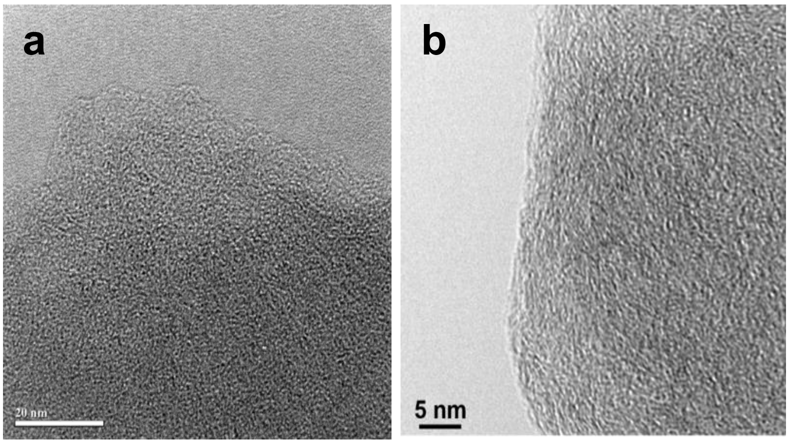

Fig. S2 TEM images of (**a**) AC, and (**b**) ACGC900

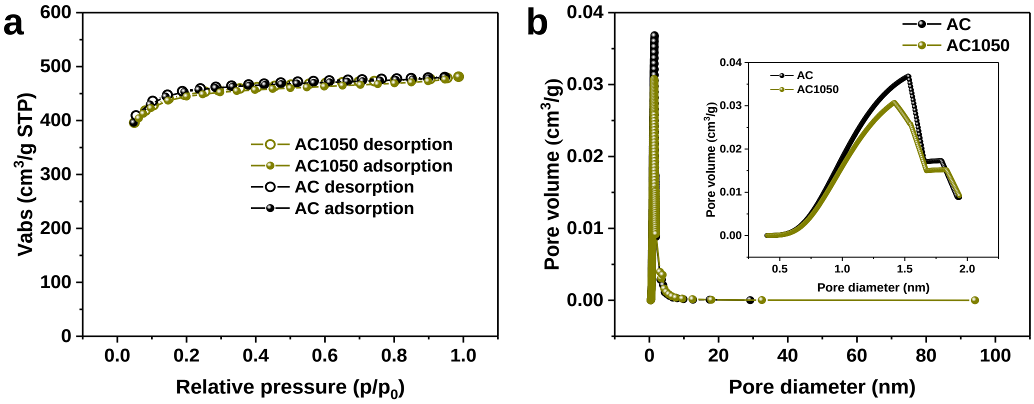

Fig. S3 (**a**) N_2 adsorption/desorption isotherms and (**b**) pore size distribution from N_2 adsorption/desorption measurement of AC and AC1050

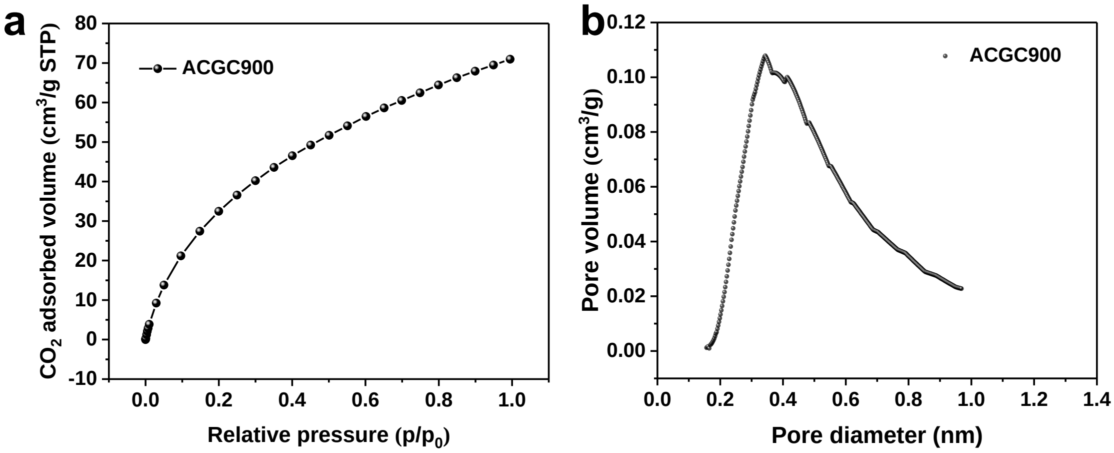

Fig. S4 (**a**) CO_2 adsorption isotherms and (**b**) the corresponding pore size distribution of ACGC900

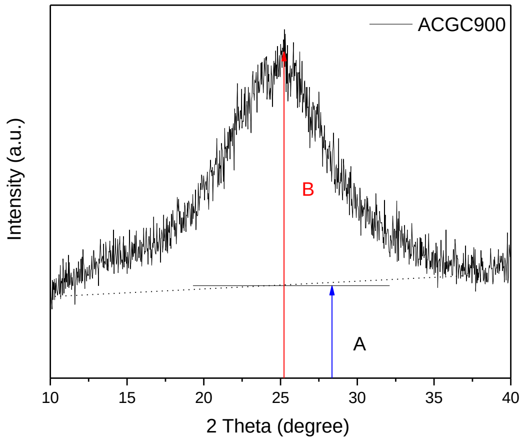

Fig. S5 Schematic representation of the definition of the parameter ***R*** used to empirically determine the degree of graphitization of the carbon samples

Notes: Figure S5 displays the method of calculating ***R*** value from the ratio of the (002) Bragg peak intensity to the background signal. ***R*** value (R = B/A) can determine the graphitization degree of the samples. A lower ***R*** value suggests a lower degree of graphitization or less stacked graphene layers.

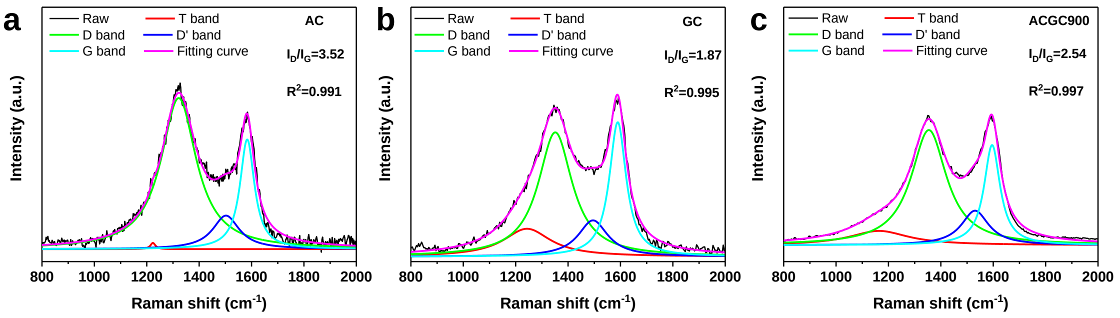

Fig. S6 Fitted Raman spectra of AC, GC and ACGC900 based on Lorentz function

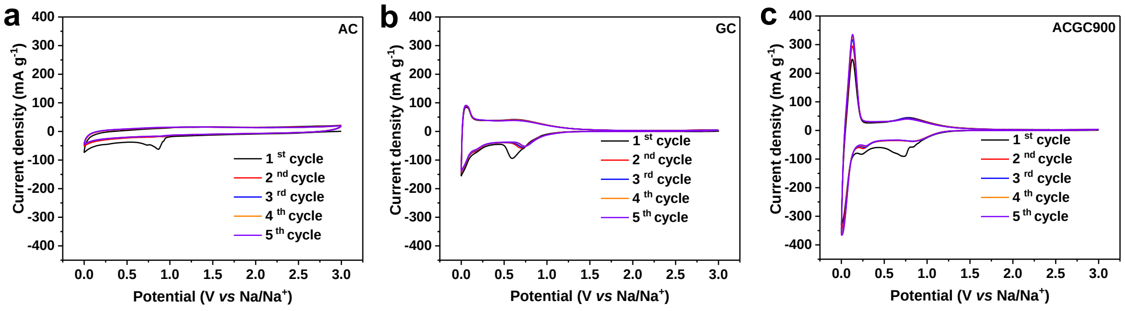

Fig. S7 First five consecutive CVs of (**a**) AC, (**b**) GC, and (**c**) ACGC900 in a voltage range of 0.001-3.0 V *vs* Na/Na^+ at a scan rate of 0.1 mV s^{-1}

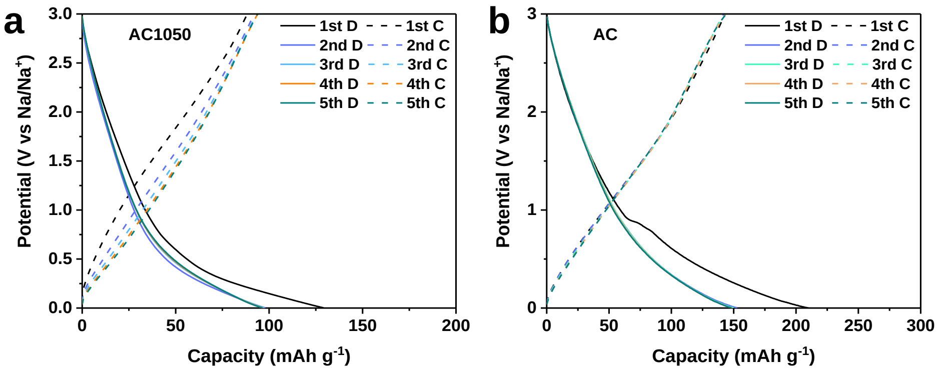

Fig. S8 Galvanostatic discharge/charge curves of (**a**) AC1050 and (**b**) AC at 50 mA g^{-1}

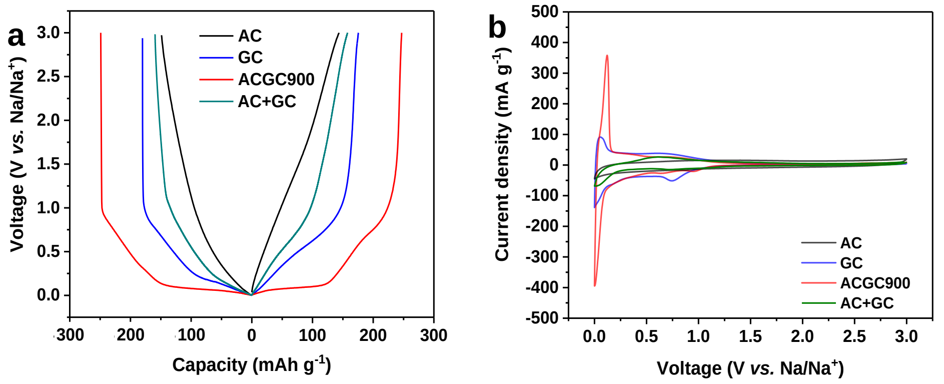

Fig. S9 (**a**) Galvanostatic discharge/charge curves at 50 mA g^{-1} and (**b**) CVs in a voltage range of 0.001-3.0 V *vs* Na/Na^{+} under 0.1 mV s^{-1} of AC, GC, ACGC900, and AC+GC

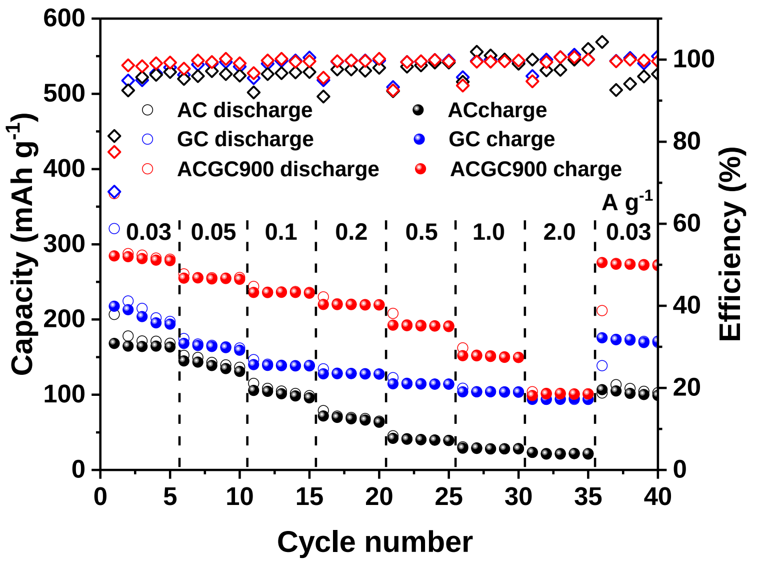

Fig. S10 Rate performance at various current densities of the AC, GC and ACGC900 electrodes

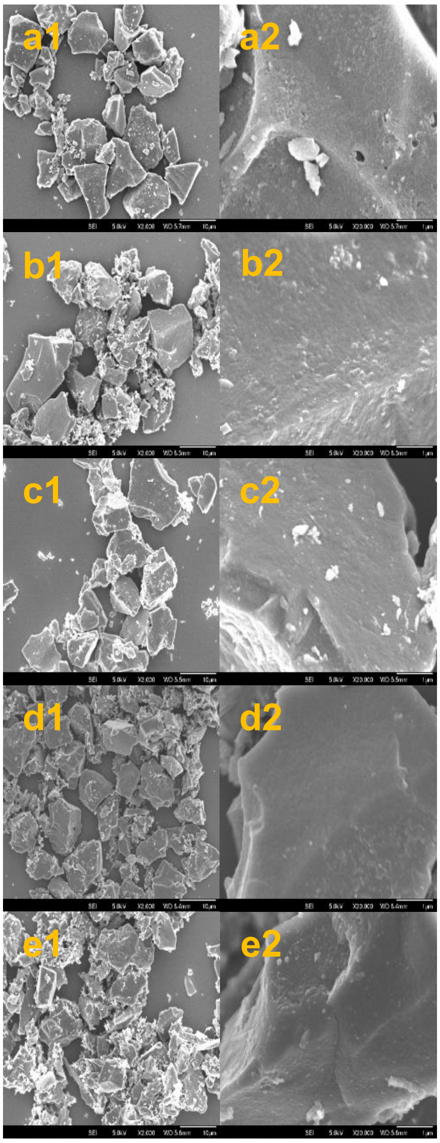

Fig. S11 (**a1-e1**) Low-resolution and (**a2-e2**) high-resolution SEM images of AC, ACGC750, ACGC900, ACGC1050, and ACGC1200, respectively (Scale bar: 10 μm for a1-e1, 1 μm for a2-e2)

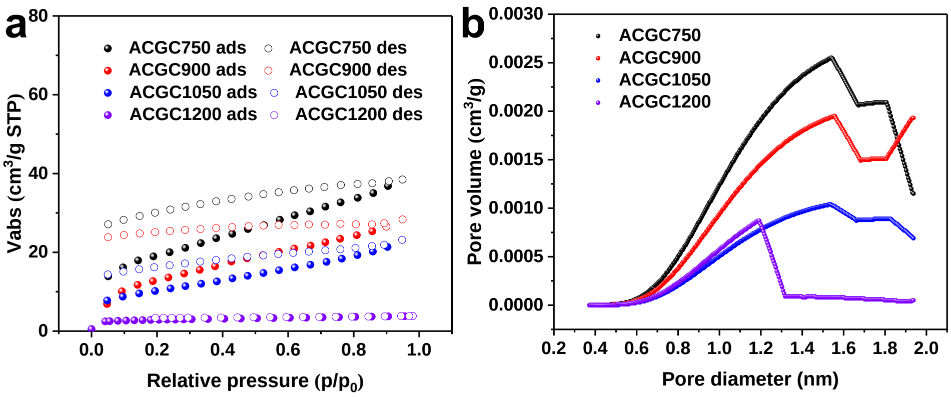

Fig. S12 N_2 adsorption/desorption isotherm and the corresponding pore size distribution of ACGCx

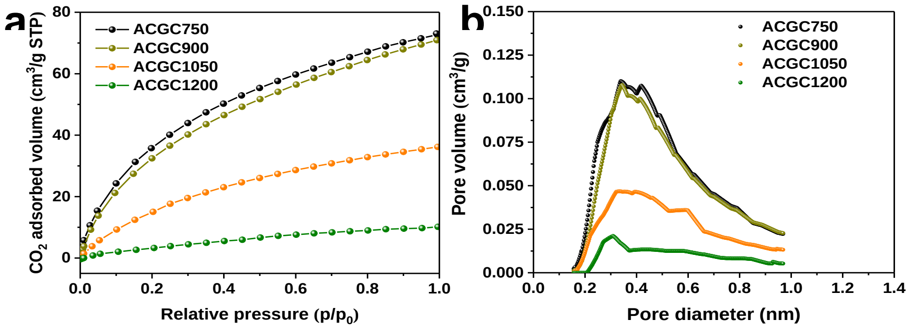

Fig. S13 CO_2 adsorption isotherm and the corresponding pore size distribution of ACGCx

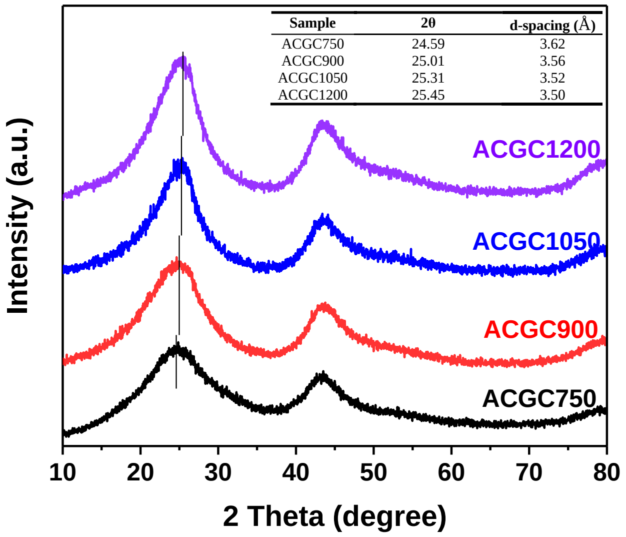

Fig. S14 XRD patterns of ACGCx (insets are the d-spacing of ACGCx)

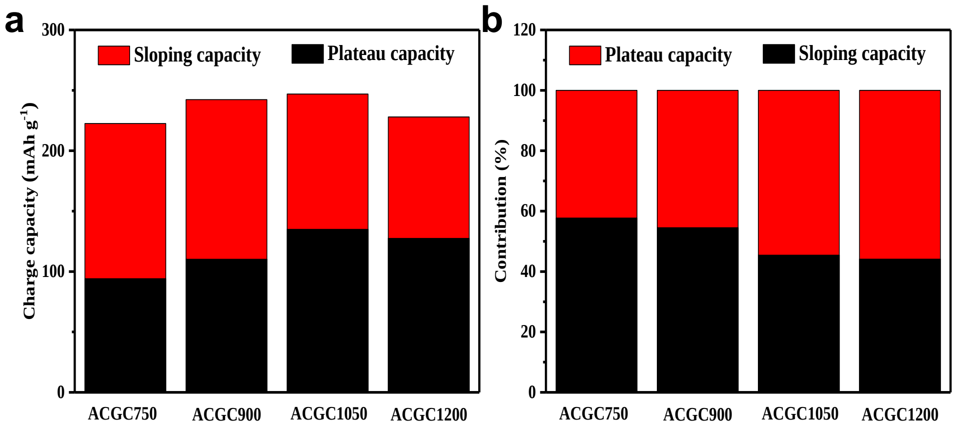

Fig. S15 (**a**) Specific capacity and (**b**) contribution of ACGCx contributed from the slope and plateau regions

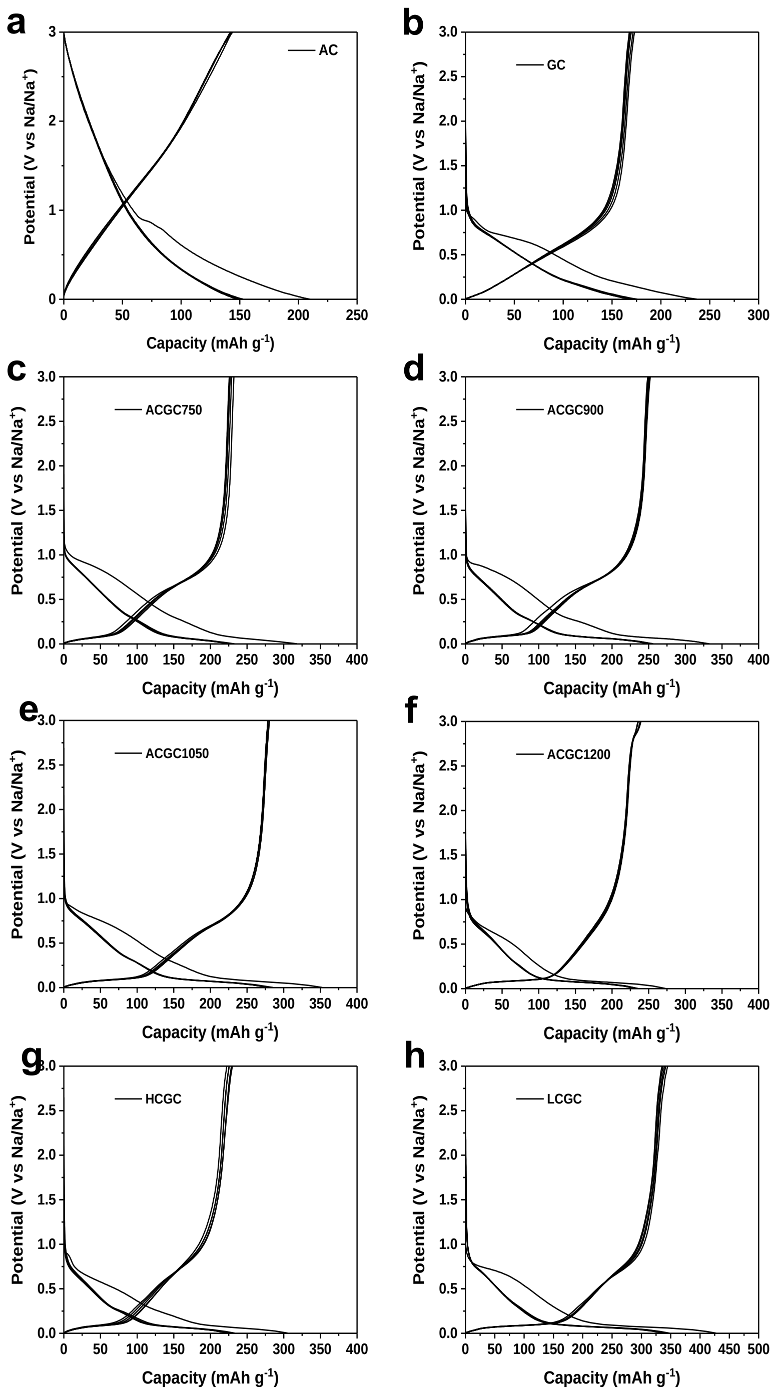

Fig. S16 First five galvanostatic discharge-charge curves of (**a**) AC, (**b**) GC, (**c**) ACGC750, (**d**) ACGC900, (**e**) ACGC1050, (**f**) ACGC1200, (**g**) HCGC, and (**h**) LCGC at 50 mA g^{-1}

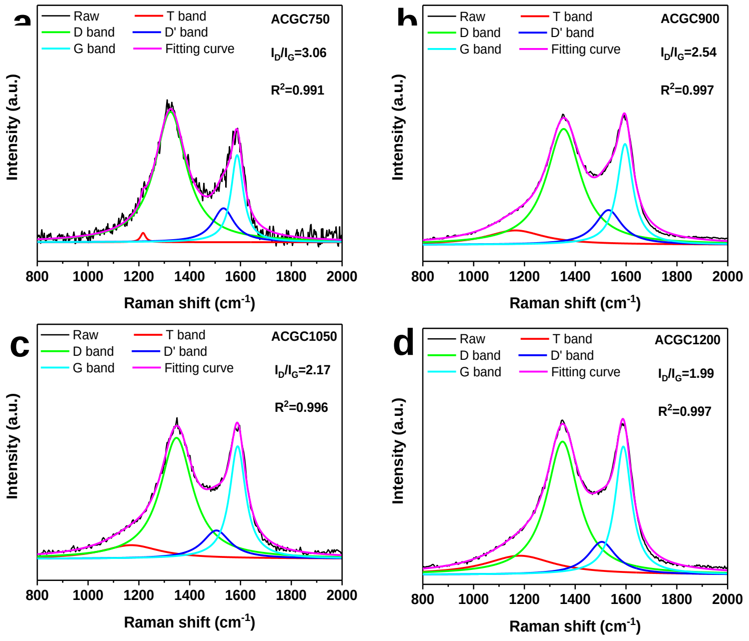

Fig. S17 Fitted Raman spectra of ACGCx based on Lorentz function

Notes: I_D/I_G ratio is calculated from the ratio between the peak area of the D band and G band in the Raman spectra. A smaller I_D/I_G ratio indicates a higher degree of graphitization.

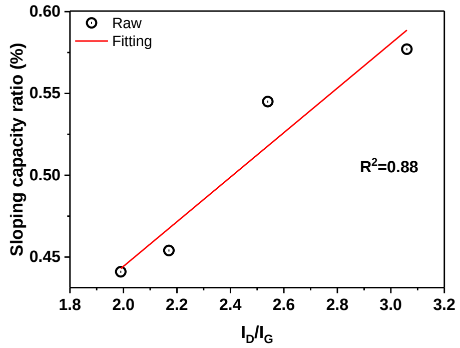

Fig. S18 Relationship between the sloping capacity contribution and the I_D/I_G ratio calculated from Raman spectra of ACGCx

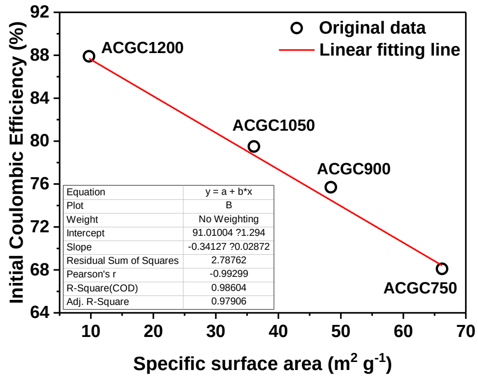

Fig. S19 Relationship between the ICE value and the BET specific surface area obtained from N_2 adsorption/desorption test

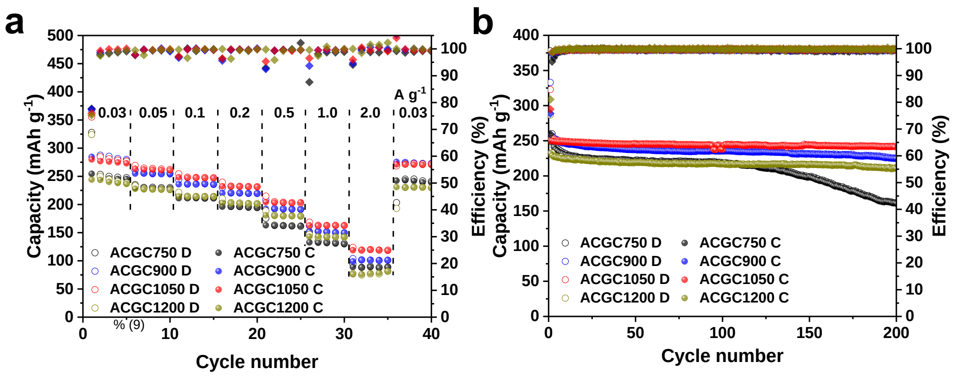

Fig. S20 (**a**) rate performance and (**b**) cycling performance of ACGCx at 50 mA g^{-1}

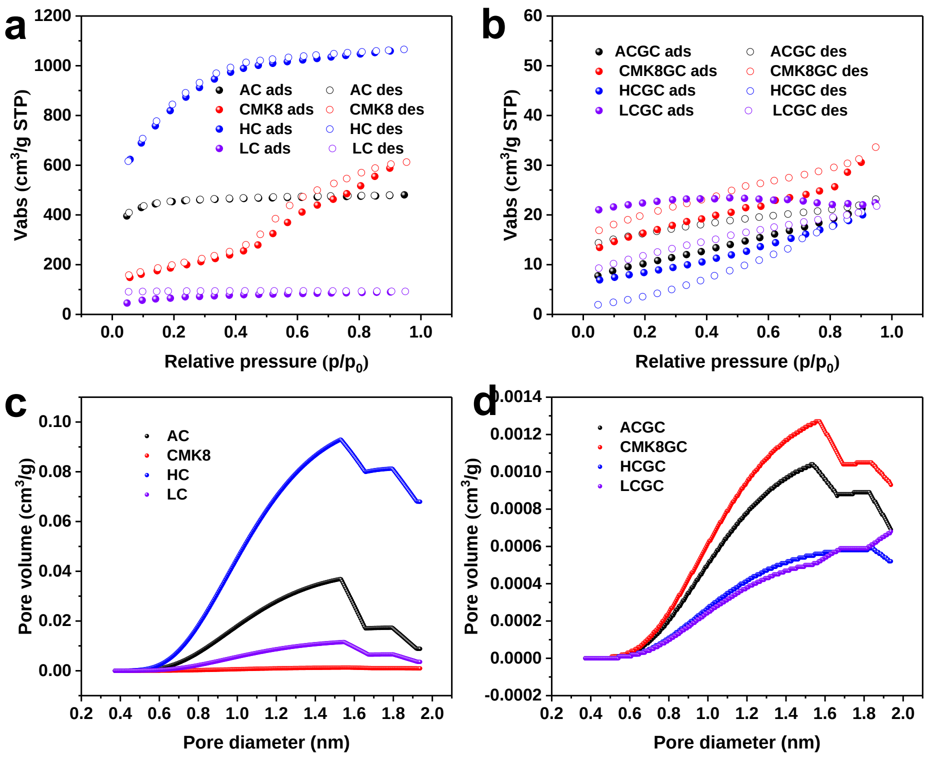

Fig. S21 (**a, b**) N_2 adsorption/desorption isotherm and (**c, d**) the corresponding pore size distribution of AC, CMK8, HC, LC, ACGC, CMK8GC, HCGC, and LCGC

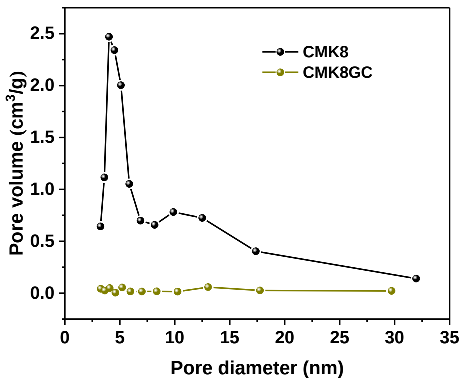

Fig. S22 The pore size distribution of CMK8 and CMK8GC

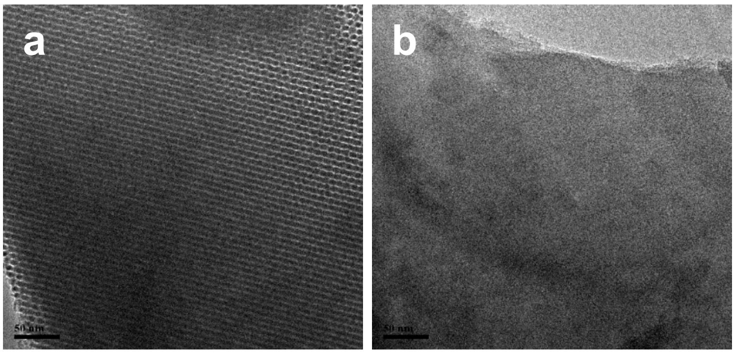

Fig. S23 The TEM images of (**a**) CMK-8 and (**b**) CMK8GC (Scale bar: 50 nm)

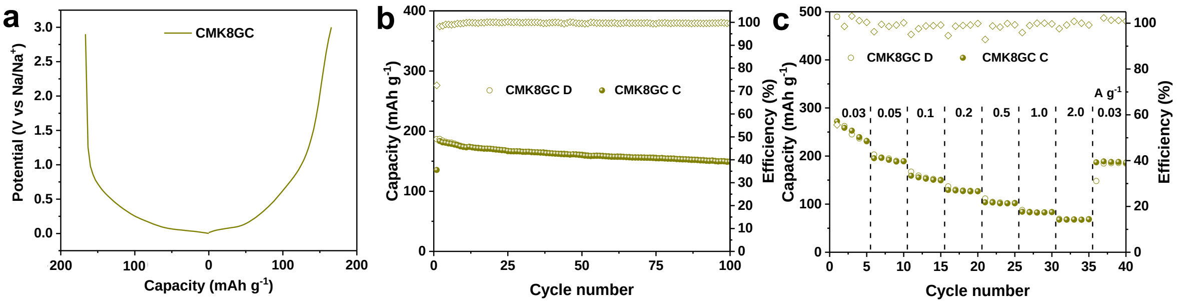

Fig. S24 (**a**) Galvanostatic discharge-charge curves at 50 mA g^{-1}, (**b**) cycling performance, and (**c**) Rate performance of CMK8GC electrodes.

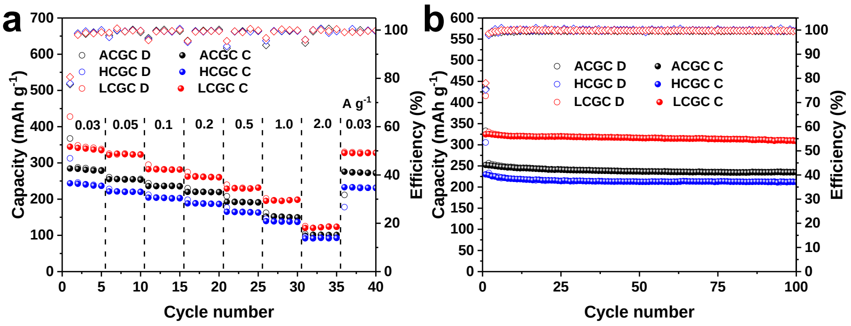

Fig. S25 (**a**) Rate performance and (**b**) cycling performance of ACGC, HCGC, LCGC electrodes at 50 mA g^{-1}

If the cell voltage is linearly proportional to $\tau^{1/2}$, the diffusion coefficient can be calculated from the GITT potential profiles by Fick's second law with the following equation:

$$\mathrm{D} = \frac{4}{\pi\tau}\left(\frac{m_B V_M}{M_B S}\right)^2\left(\frac{\Delta E_s}{\Delta E_\tau}\right)^2$$

The density of carbon was calculated according to the following equation:

$$\rho = \frac{1}{V_{\text{total}} + \frac{1}{\rho_{carbon}}}$$

where ρ (g cm^{-3}) is the density of carbon, V_{total} (cm^3 g^{-1}) is the total pore volume measured from the N_2 isotherm, ρ_{carbon} is the true density of carbon (2.08 g cm^{-3}).

For the GITT tests, the cell was discharged/charged at C/10 with a current pulse duration of 0.5 h and an interval of 1 h [S1, S2].

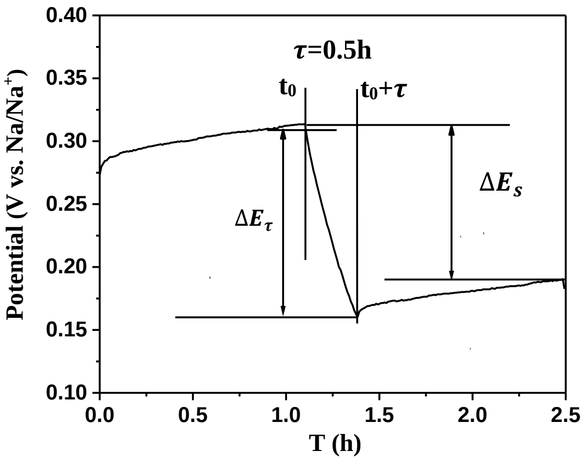

Fig. S26 Current step diagram at 0.304 V *vs* Na/Na^+ of third sodiation process of ACGC electrode for sodium ion batteries

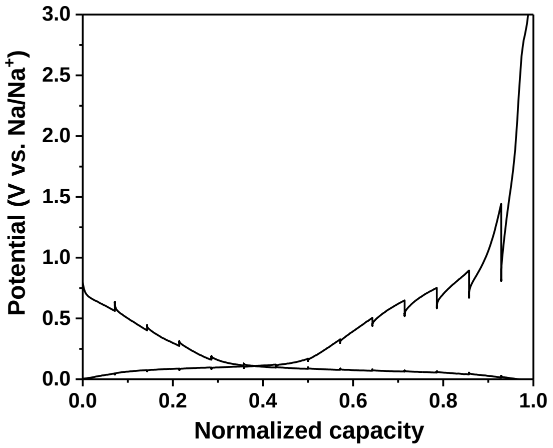

Fig. S27 GITT potential profiles of ACGC

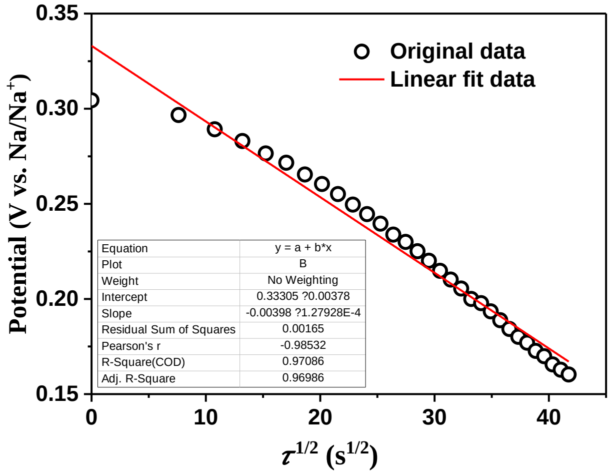

Fig. S28 Linear behaviour of the potential *vs* $\tau^{1/2}$ in GITT at (A) 0.304 V *vs* Na/Na^+ of ACGC during the third sodiation process

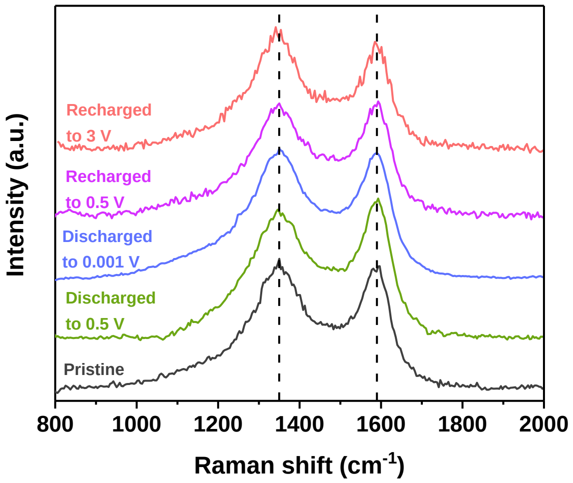

Fig. S29 *Ex-situ* Raman spectra of the carbon electrode at different discharge/charge states

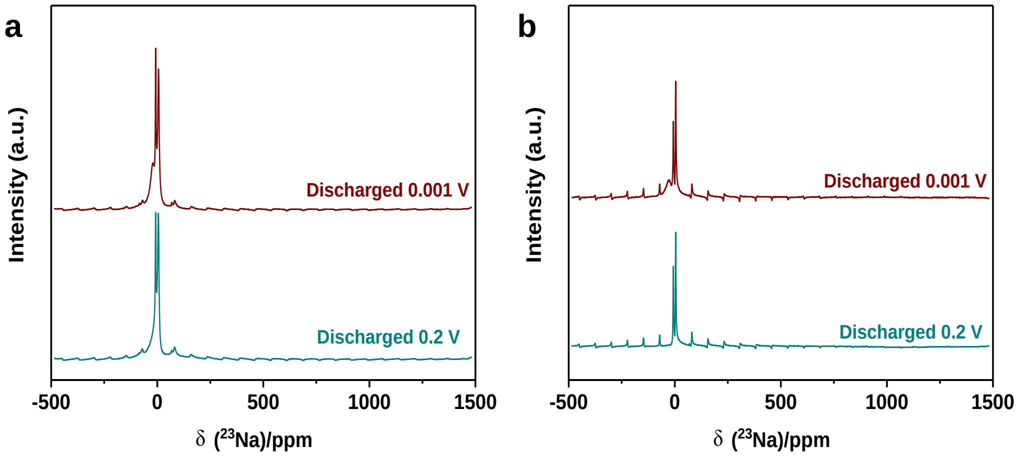

Fig. S30 The *ex-situ* ^{23}Na solid-state NMR spectra for two groups of carbon electrodes at different potentials: (**a**) the first group and (**b**) the second group

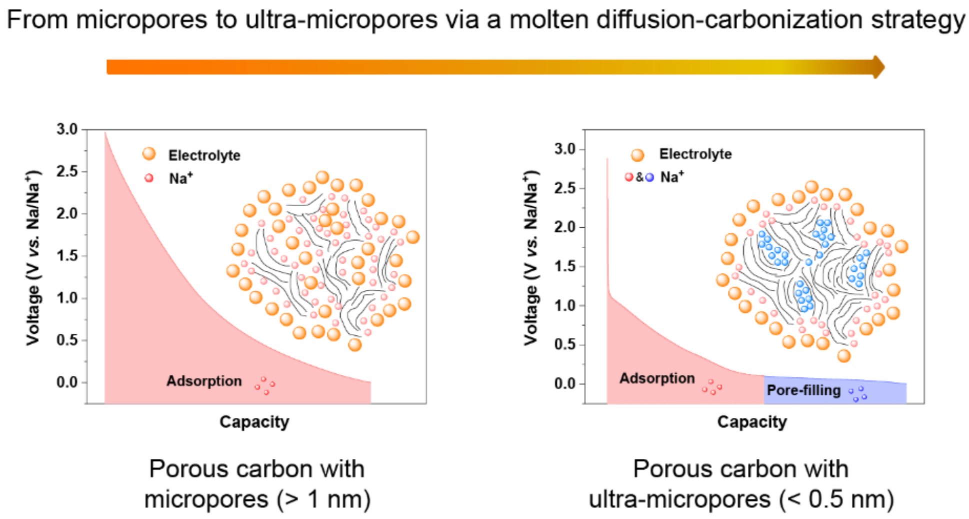

Fig. S31 Schematic illustration of the proposed sodium-ion storage mechanism

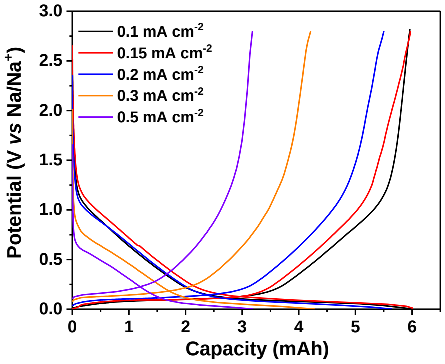

Fig. S32 Discharge-charge profiles of the LCGC thick electrode at different current densities

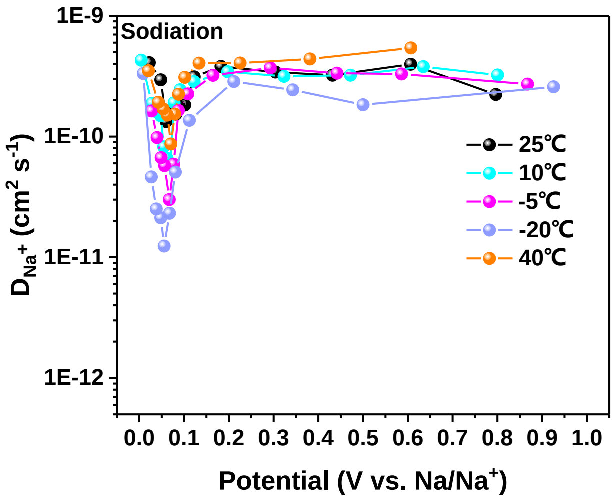

Fig. S33 D_{Na^+} values calculated from GITT tests during the discharge-charge process of the thick LCGC electrode at various temperatures

Table S1 Specific surface area and volume of ACGCx calculated from N_2 adsorption/ desorption tests

Sample	S_{BET} [m^2 g^{-1}]	$S_{micropore}$ [m^2 g^{-1}][a]	$S_{external}$ [m^2 g^{-1}][b]	$V_{micropore}$ [cm^3 g^{-1}][c]	$V_{external}$ [cm^3 g^{-1}][d]	V_{total} [cm^3 g^{-1}][e]
AC	1429	1364	65	0.691	0.700	1.391
AC1050	1322	1258	64	0.671	0.723	1.394
AC-PTCDA	29.6	5.2	24.4	0.012	0.034	0.046
ACGC750	66.2	42.6	23.6	0.028	0.090	0.118
ACGC900	48.4	32.8	15.6	0.019	0.065	0.084
ACGC1050	36.1	20.6	15.6	0.015	0.054	0.069
ACGC1200	9.7	8.1	1.6	0.004	0.007	0.011

[a] Micropore specific surface area determined by the t-method by N_2 adsorption branch at 77 K.

[b] External specific surface area determined by the t-method external surface area

[c] Micropore volume detemined by the HK method by N_2 adsorption branch at 77 K

[d] External volume determined by the summation of mesopore volume from the DFT method and macropore volume calculated from adsorption curve by BJH method

[e] Total volume determined by the summation of the micropore, the external volume

Table S2 Specific surface area and volume of AC and AC1050

Sample	S_{BET} [m^2 g^{-1}]	$S_{micropore}$ [m^2 g^{-1}][a]	$S_{external}$ [m^2 g^{-1}][b]	$V_{micropore}$ [cm^3 g^{-1}][c]	$V_{external}$ [cm^3 g^{-1}][d]	V_{total} [cm^3 g^{-1}][e]
AC	1429	1364	65	0.691	0.700	1.391
AC1050	1322	1258	64	0.671	0.723	1.394

[a] Micropore specific surface area determined by the t-method by N_2 adsorption branch at 77 K.

[b] External specific surface area determined by the t-method external surface area

[c] Micropore volume detemined by the HK method by N_2 adsorption branch at 77 K

[d] External volume determined by the summation of mesopore volume from the DFT method and macropore volume calculated from adsorption curve by BJH method

[e] Total volume determined by the summation of the micropore, the external volume

Table S3 Specific surface area for ACGCx calculated from CO_2 and N_2 adsorption/desorption tests

Sample	S^{N2}_{BET} [m^2 g^{-1}]	S^{CO2}_{BET} [m^2 g^{-1}]	V^{N2}_{total} [cm^3 g^{-1}]	V^{CO2}_{total} [cm^3 g^{-1}]	Skeletal density [g cm^{-3}]
ACGC750	66.2	201.4	0.118	0.369	1.89
ACGC900	48.4	196.6	0.084	0.365	1.98
ACGC1050	36.1	99.9	0.069	0.185	2.08
ACGC1200	9.7	18.6	0.011	0.096	2.14

Table S4 Calculated ***R*** value of various samples

**Sample**	***R* value**
ACGC750	2.93
ACGC900	3.54
ACGC1050	4.09
ACGC1200	4.23
GC	5.77
AC	1.61

Table S5 Initial coulombic efficiency (ICE) value of various samples

Sample	ICE (%)
ACGC750	73.0
ACGC900	75.7
ACGC1050	79.5
ACGC1200	87.9
HCGC	75.3
LCGC	80.6
GC	73.3
AC	68.1

Table S6 Specific surface area and volume calculated from N_2 adsorption/desorption tests

Sample	S_{BET} [m^2 g^{-1}]	$S_{micropore}$ [m^2 g^{-1}][a]	$S_{external}$ [m^2 g^{-1}][b]	$V_{micropore}$ [cm^3 g^{-1}][c]	$V_{external}$ [cm^3 g^{-1}][d]	V_{total} [cm^3 g^{-1}][e]
CMK8	668	0	668	0.274	1.749	2.023
LC	224	199	25	0.097	0.170	0.267
AC	1429	1364	65	0.691	0.700	1.391
HC	2939	2455	484	1.197	1.723	2.920
CMK8GC	55.6	32.6	23	0.024	0.074	0.098
LCGC	19.4	4.3	15.1	0.005	0.06	0.065
ACGC	36.1	20.6	15.6	0.015	0.054	0.069
HCGC	29	11	18	0.012	0.053	0.065

[a] Micropore specific surface area determined by the t-method by N_2 adsorption branch at 77 K.

[b] External specific surface area determined by the t-method external surface area

[c] Micropore volume detemined by the HK method by N_2 adsorption branch at 77 K

[d] External volume determined by the summation of mesopore volume from the DFT method and macropore volume calculated from adsorption curve by BJH method

[e] Total volume determined by the summation of the micropore, the external volume

Table S7 Specific surface area (calculated from N_2 adsorption/desorption tests), filler/host ratio and skeletal density for LCGC, ACGC, and HCGC

Sample	S^{N2}_{BET} [m^2 g^{-1}]	V^{N2}_{total} [cm^3 g^{-1}][e]	filler/host ratio	Skeletal density [g cm^{-3}]
LCGC	19.4	0.065	0.45	2.02
ACGC	36.1	0.069	2.36	2.08
HCGC	29	0.065	4.96	2.23

Table S8 A survey of hard carbon anode for SIBs

**Anode material**	**Reversible capacity (mAh g^{-1} @ mA g^{-1})**	**Cyclability (capacity retention rate (%) @ mA g^{-1} @ cycle number)**	**Rate capacity (mAh g^{-1} @ mA g^{-1})**	**Carbonization Temperature (°C) @ ICE (%)**
Hollow carbon nanowires [S3]	251@50	82@50@400	149@500	1150@50.5
G-HC [S4]	281@20	97@20@195	~20@1000	1100@78
P-doped hard carbon [S5]	359@20	96@20@60	~50@1000	1100@73
Hard carbon with low defects/surface area [S6]	338@20	93@20@100	NA	1300@78.9
Hard carbon paper [S7]	338@20	84@200@1000	170@2000	1300@91.2
Waste cork-derived hard carbon [S8]	358@30	87@30@200	~175@600	1600@81
Phenolformaldehyde resin-derived hard carbon [S9]	410@30	93@30@40	~25@600	1550@83
P-doped hard carbon [S10]	310@20	92@20@100	NA	1300@85
Hard carbon [S11]	254@30	89@20@150	162@300	1400@82
Hard carbon [S12]	284@30	94@30@100	90@600	1400@88

Supplementary References

[S1] J. Yang, S. Xiao, X. Cui, W. Dai, X. Lian et al., Inorganic-anion-modulated synthesis of 2D nonlayered aluminum-based metal-organic frameworks as carbon precursor for

capacitive sodium ion storage. Energy Storage Mater. **26**, 391-399 (2020). https://doi.org/10.1016/j.ensm.2019.11.010

[S2] B. Cao, Q. Zhang, H. Liu, B. Xu, S. Zhang et al., Graphitic carbon nanocage as a stable and high power anode for potassium-ion batteries. Adv. Energy Mater. 1801149 (2018). https://doi.org/10.1002/aenm.201801149

[S3] Y. Cao, L. Xiao, M.L. Sushko, W. Wang, B. Schwenzer, et al., Sodium ion insertion in hollow carbon nanowires for battery applications. Nano Lett. **12**(7), 3783-3787 (2012). https://doi.org/10.1021/nl3016957

[S4] W. Luo, C. Bommier, Z. Jian, X. Li, R. Carter et al., Low-surface-area hard carbon anode for na-ion batteries via graphene oxide as a dehydration agent. ACS Appl. Mater. Interfaces **7**(4), 2626-2631 (2015). https://doi.org/10.1021/am507679x

[S5] Z. Li, L. Ma, T.W. Surta, C. Bommier, Z. Jian et al., High capacity of hard carbon anode in na-ion batteries unlocked by pox doping. ACS Energy Lett. **1**(2), 395-401 (2016). https://doi.org/10.1021/acsenergylett.6b00172

[S6] L. Xiao, H. Lu, Y. Fang, M.L. Sushko, Y. Cao et al., Low-defect and low-porosity hard carbon with high coulombic efficiency and high capacity for practical sodium ion battery anode. Adv. Energy Mater. **8**(20), 1703238 (2018). https://doi.org/10.1002/aenm.201703238

[S7] B.H. Hou, Y.Y. Wang, Q.L. Ning, W.H. Li, X.T. Xi et al., Self-supporting, flexible, additive-free, and scalable hard carbon paper self-interwoven by 1d microbelts: Superb room/low-temperature sodium storage and working mechanism. Adv. Mater. e1903125 (2019). https://doi.org/10.1002/adma.201903125

[S8] Y. Li, Y. Lu, Q. Meng, A.C.S. Jensen, Q. Zhang et al., Regulating pore structure of hierarchical porous waste cork-derived hard carbon anode for enhanced na storage performance. Adv. Energy Mater. 1902852 (2019). https://doi.org/10.1002/aenm.201902852

[S9] Q. Meng, Y. Lu, F. Ding, Q. Zhang, L. Chen et al., Tuning the closed pore structure of hard carbons with the highest na storage capacity. ACS Energy Lett. 2608-2612 (2019). https://doi.org/10.1021/acsenergylett.9b01900

[S10] D. Sun, B. Luo, H. Wang, Y. Tang, X. Ji et al., Engineering the trap effect of residual oxygen atoms and defects in hard carbon anode towards high initial coulombic efficiency. Nano Energy. **64**, 103937 (2019). https://doi.org/10.1016/j.nanoen.2019.103937

[S11] Y. Li, Y.-S. Hu, H. Li, L. Chen, X. Huang, A superior low-cost amorphous carbon anode made from pitch and lignin for sodium-ion batteries. J. Mater. Chem. A **4**(1), 96-104 (2016). https://doi.org/10.1039/C5TA08601A

[S12] Y. Li, L. Mu, Y.-S. Hu, H. Li, L. Chen et al., Pitch-derived amorphous carbon as high performance anode for sodium-ion batteries. Energy Storage Materials **2**, 139-145 (2016). https://doi.org/10.1016/j.ensm.2015.10.003

[S13] Y. Lu, C. Zhao, X. Qi, Y. Qi, H. Li et al., Pre-oxidation-tuned microstructures of carbon anodes derived from pitch for enhancing na storage performance. Adv. Energy Mater. **8**(27), 1800108 (2018). https://doi.org/10.1002/aenm.201800108
